# Low-dose TNF augments fracture healing in normal and osteoporotic bone by up-regulating the innate immune response

**DOI:** 10.15252/emmm.201404487

**Published:** 2015-03-14

**Authors:** James K Chan, Graeme E Glass, Adel Ersek, Andrew Freidin, Garry A Williams, Kate Gowers, Ana I Espirito Santo, Rosemary Jeffery, William R Otto, Richard Poulsom, Marc Feldmann, Sara M Rankin, Nicole J Horwood, Jagdeep Nanchahal

**Affiliations:** 1 Kennedy Institute of Rheumatology, University of OxfordOxford, UK; 2 National Heart and Lung Institute, Imperial College LondonLondon, UK; 3 Histopathology Laboratory and In Situ Hybridisation Service, Cancer Research UK – London Research InstituteLondon, UK

**Keywords:** bone, CCL2, fracture, inflammation, TNF

## Abstract

The mechanism by which trauma initiates healing remains unclear. Precise understanding of these events may define interventions for accelerating healing that could be translated to the clinical arena. We previously reported that addition of low-dose recombinant human TNF (rhTNF) at the fracture site augmented fracture repair in a murine tibial fracture model. Here, we show that local rhTNF treatment is only effective when administered within 24 h of injury, when neutrophils are the major inflammatory cell infiltrate. Systemic administration of anti-TNF impaired fracture healing. Addition of rhTNF enhanced neutrophil recruitment and promoted recruitment of monocytes through CCL2 production. Conversely, depletion of neutrophils or inhibition of the chemokine receptor CCR2 resulted in significantly impaired fracture healing. Fragility, or osteoporotic, fractures represent a major medical problem as they are associated with permanent disability and premature death. Using a murine model of fragility fractures, we found that local rhTNF treatment improved fracture healing during the early phase of repair. If translated clinically, this promotion of fracture healing would reduce the morbidity and mortality associated with delayed patient mobilization.

## Introduction

Bone fractures are a very common clinical problem affecting of 3.6% of the UK population every year (Donaldson *et al*, 2008). While the majority of fractures heal uneventfully, they are particularly problematic in certain subgroups of patients, including high-energy open fractures which represent limb-threatening injuries prone to delayed and non-union, and fractures in osteoporotic bone, which represent the greatest unmet clinical need. Although addition of exogenous bone morphogenetic proteins (BMPs) results in improved healing in animal models, clinical trials of BMPs for tibial non-union (Friedlaender *et al*, 2001) and fracture healing (Govender *et al*, 2002) have failed to achieve the efficacy anticipated (Lane, 2001; Lieberman *et al*, 2002). This is probably because a single supraphysiological dose of BMP does not induce the complex pattern of growth factor and cytokine production required for optimal fracture repair. Currently, there is no approved therapy to enhance healing of fragility fractures in osteoporotic bone (Kanakaris *et al*, 2009). Antibodies to endogenous inhibitors of the Wnt/β-catenin signaling pathway, including sclerostin and dickkopf-1, have shown early promise but are limited to systemic administration, leading to non-specific bone deposition. Furthermore, these are associated with safety concerns, including carcinogenesis and closure of osteal foramina (Baron & Hesse, 2012). Therefore, there is an urgent unmet medical need to develop therapeutic strategies to improve local bone regeneration and the healing of fractures.

The ideal biological therapy for accelerating fracture healing would entail local administration of pro-osteogenic factor(s) at the time of surgical treatment. Therefore, it is critical to understand how the early inflammatory response initiates and orchestrates fracture repair, an area that remains poorly understood. A competent immune system is essential for effective wound healing, although excessive inflammation has been found to be deleterious. For example, the absence of TNF or IL-6 signaling has been found to impair fracture healing (Gerstenfeld *et al*, 2003a; Yang *et al*, 2007), whereas persistent and elevated levels of TNF in rheumatoid arthritis are associated with bone destruction (Binder *et al*, 2013) and systemic daily administration of high-dose TNF in a rat rib fracture model also resulted in impaired healing (Hashimoto *et al*, 1989). Therefore, there is a critical balance to ensure an optimal healing environment (Chan *et al*, 2012c).

It is currently unknown how early inflammation initiates the process of fracture healing. We have previously shown that injection of low-dose rhTNF at the fracture site in a murine tibial fracture model of endochondral healing led to improved callus formation and earlier remodeling (Glass *et al*, 2011). This was in part achieved through the recruitment and osteogenic differentiation of mesenchymal stromal cells (MSCs) by TNF (Glass *et al*, 2011). Studies of early fracture hematoma by other groups have shown that the inflammatory phase following fracture is critical to recruit cells and orchestrate the events necessary for fracture healing (Grundnes & Reikeras, 1993; Kolar *et al*, 2010, 2011; Hoff *et al*, 2013).

Here, we describe the early fracture-healing pathway and the enhancement of the early innate immune response by TNF administration in the first 24 h after injury. Interventions targeting events during this early time window should allow effective and simple clinical translation, as the therapeutic would be administered at the time of reduction and stabilization of the fractures.

## Results

### Local addition of TNF during early inflammatory phase augments fracture repair in C57BL/6 mice

**Figure 1:**
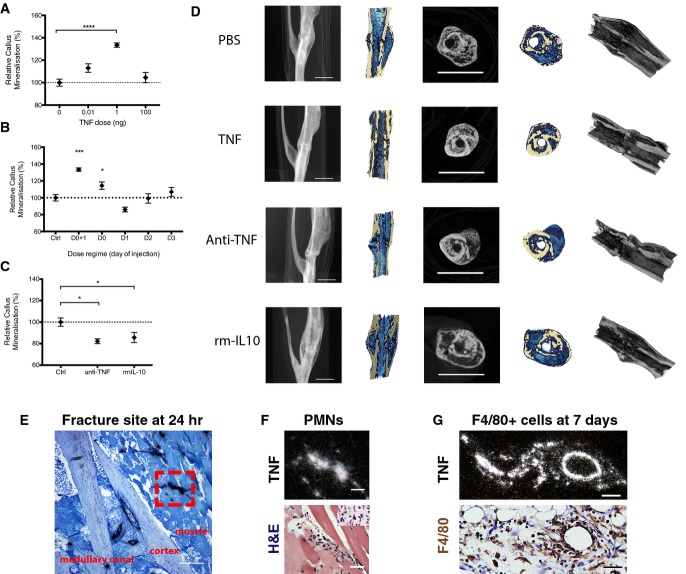
Role of TNF in fracture healing *in vivo*

We previously reported that 1 ng rhTNF injected at the fracture site on days 0 and 1 improved fracture repair (Glass *et al*, 2011). To identify the optimal dose *in vivo*, we injected increasing amounts of rhTNF at the fracture site on days 0 and 1 (i.e. immediately and at 24 h) following osteotomy and found that rhTNF was effective in augmenting fracture healing, as indicated by increased relative % callus mineralization, by day 28 after injury only at 1 ng (Fig1A). As shown by the micro-CT reconstructions, treatment with 1 ng of rhTNF led to improved healing and earlier remodeling as evidenced by a more compact and mineralized fracture callus (Fig1D).

Addition of rhTNF at the fracture site on days 0 and 1 led to augmented healing, indicated by increased % callus mineralization, at day 28 after operation in a dose-dependent manner. Data are presented as mean ± SEM. Treatment with 1 ng TNF versus PBS control, ****P < 0.0001 by one-way ANOVA with Dunnett's multiple comparisons test.

Addition of rhTNF at the fracture site must be given within the first 24 h to augment healing, indicated by % callus mineralization. Data are presented as mean ± SEM. 1 ng TNF versus PBS control treatment on days 0 and 1, ***P = 0.0009 by one-way ANOVA with Dunnett's multiple comparisons test.

Treatment with systemic anti-TNF or local rmIL-10 led to impaired fracture repair at day 28, indicated by % callus mineralization. Data are presented as mean ± SEM. Treatment with PBS control versus anti-TNF, *P = 0.037, PBS control versus rmIL-10, *P = 0.037, by unpaired two-sided *t*-tests.

Representative micro-CT images at the fracture site showing (from left to right): lateral view of tibia, cross-section, and cross and longitudinal sections with color overlay and 3D reconstruction. In the color overlays, the shade of blue corresponds to percentage mineralization: light blue denotes soft immature callus, whereas dark blue denotes hard mineralized callus. Scale bar, 2 mm.

Representative ISH image (light field) showing TNF expression at the murine fracture site at 24 h after fracture. Scale bar, 250 μm. Red box indicates region of interest.

High-power micrographs of region of interest from (E): at 24 h after fracture, mTNF expression (white signal on dark field, above) co-localized with polymorphonuclear cells found on the adjacent H&E section (below). Scale bar, 25 μm. Neutrophils were identified by their polymorphonuclear morphology as well as positive dark brown staining with anti-neutrophil elastase.

High-power micrographs: at 7 days, TNF expression (white signal on dark field, above) co-localized with F4/80-positive cells (stained dark brown) extravasating from a blood vessel on the adjacent H&E section (below). Scale bar, 25 μm.

We also found that augmented fracture healing was only evident when rhTNF at 1 ng was administered at the fracture site over the first 24 h (Fig1B). This would be particularly relevant for clinical translation as this would enable treatment at the time of reduction and stabilization of the fracture.

### Inhibition of TNF impairs fracture repair *in vivo*

To confirm the role of TNF in fracture healing *in vivo*, we inhibited endogenous TNF by either systemic administration of a neutralizing antibody to TNF, TN3, or local injection of recombinant murine IL-10 (rmIL-10), which also inhibits TNF expression and release (Smallie *et al*, 2010), at the fracture site. As shown in Fig1C, the relative callus mineralization was reduced by both treatments at day 28. The micro-CT reconstructions showed that systemic anti-TNF led to poor bridging across the fracture site and an incomplete and poorly mineralized callus, whereas local rmIL-10 treatment led to a large, unmineralized callus, the hallmarks of an immature callus (Fig1D). These observations are consistent with the augmented callus maturation seen in mice treated with 1 ng of TNF.

### TNF is expressed during the early inflammatory response by innate immune cells

TNF is released as part of the early inflammatory response to the bone injury as it is for other injuries and stresses (Kon *et al*, 2001) and is an important mediator in fracture repair (Gerstenfeld *et al*, 2003a; Glass *et al*, 2011). However, the precise quantities, and spatial and temporal sequences of release as well as its role in fracture healing remain unclear. We found that circulating serum levels of TNF did not differ significantly from no injury controls up to 72 h post-fracture (Supplementary Fig S1A), indicating that the level of trauma induced by the isolated tibial fracture in our murine model is insufficient to lead to a systemic inflammatory response. To assess the local cytokine environment at the fracture site, murine fracture supernatants were produced by incubating fractured tibiae in media as described previously (Glass *et al*, 2011). Consistent with our published findings in human fracture supernatants (Glass *et al*, 2011), the levels of TNF were very low (< 2 pg/ml, the level of detection of the MSD chemiluminescent system).

**Figure 2:**
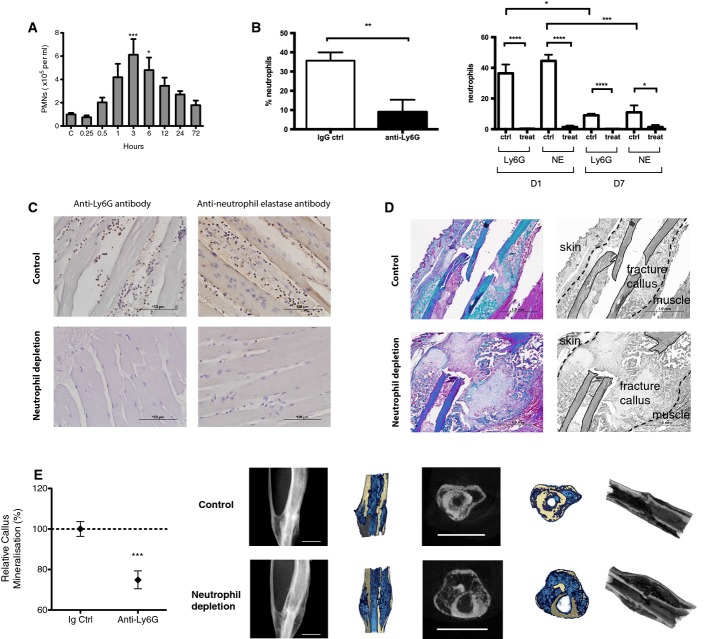
Role of neutrophils in fracture healing

Our observation that TNF inhibition impaired fracture healing *in vivo* suggests that endogenous TNF is expressed locally, albeit at a low level. Hence, we employed *in situ* hybridization on histological sections of the murine fracture site (Fig1E–G) to enable identification of the cellular sources of TNF *in vivo*. We found that TNF was expressed within 15 min of injury, co-localizing first with endothelial cells and neutrophils (Fig1F). The neutrophils were identified by their polymorphonuclear morphology as well as by positive staining with anti-neutrophil elastase or anti-Ly6G (Fig2B). From day 3 onwards, TNF expression co-localized with cells of the monocyte/macrophage lineage (F4/80^+^) (Fig1G). Neutrophils were the predominant cell type present before day 3 (at 3 h, 24 h, and 3 days) with no F4/80^+^ cells identified at this stage, while the latter were the predominant cell type after day 3 (day 5 and day 7) as shown by the representative sections in Fig1G.

Neutrophils were mobilized into the murine systemic circulation within 30 min of injury in our murine fracture model. Compared to control and number of blood neutrophils at 3 h ***P = 0.0006 and at 6 h *P = 0.042, by one-way ANOVA with Dunnett correction.

Depletion of neutrophils using anti-Ly6G. Intravenous anti-Ly6G treatment led to depletion of neutrophils in the systemic circulation (left) as well the local peri-fracture soft tissues (right). Left: counts of neutrophils in the blood harvested by cardiac puncture at 3 h post-injury. Data are presented as mean ± SEM (**P = 0.0074 using unpaired one-tailed *t*-test). Right: counts of positively stained infiltrative neutrophils in the adjacent muscle to the fracture site comparing neutrophil depletion with anti-Ly6G antibody versus IgG control at day 1 and day 7 post-fracture. At day 1, number of neutrophils in control group as detected by Ly6G and NE in control versus treatment: ****P = 0.0001 for both Ly6G and NE, by unpaired two-tailed *t*-test. At day 7, number of neutrophils in control group as detected by Ly6G and NE in control versus treatment groups: ****P = 0.0001 for Ly6G and *P = 0.020 for NE, by unpaired two-tailed *t*-test. Number of neutrophils at day 1 versus day 7 in control groups, *P = 0.037 for Ly6G and ***P = 0.0006 for NE, by unpaired two-tailed *t*-test.

Representative sections showing local infiltration of neutrophils in the adjacent muscle stained using anti-Ly6G and anti-neutrophil elastase primary antibodies at day 1 post-fracture following treatment with anti-Ly6G antibody or IgG control. Scale bar, 100 μm.

Depletion of neutrophils using anti-Ly6G led to impaired fracture healing. Representative section stained with Masson's trichrome at day 14 comparing fracture healing in mouse treated with isotype control (top) versus anti-Ly6G (bottom). Black and white images in the right column are identical to the color images in the left column; they have been labeled to clearly demonstrate the anatomical structures. Control section shows advanced mineralized callus, while treatment section shows a large immature unmineralized callus. Muscle fibers: red; collagen, bone, and mineralized callus: green. Scale bar, 1 mm.

Anti-Ly6G treatment led to impaired fracture healing as shown by the reduced % callus mineralization at day 28 after surgery and representative micro-CT images. Anti-Ly6G treatment led to delayed mineralization and remodeling of the fracture callus compared to control. Data are presented as mean ± SEM. ***P = 0.0006 by unpaired two-sided *t*-test. Scale Bar, 2 mm.

### Neutrophil recruitment occurs following fracture and promotes fracture repair

As recruitment of neutrophils represents a key early event during the inflammatory response (Nathan, 2006), we investigated their role in fracture healing. First, we assessed the systemic mobilization of neutrophils in our murine fracture model. Blood neutrophil count increased within 30 min of injury and peaked at 3 h following fracture (Fig2A).

Next, mice were treated with a validated and specific Ly6G-blocking antibody (Daley *et al*, 2008). This effectively inhibited the mobilization of neutrophils into the systemic circulation as well as the local recruitment of neutrophils to the fracture site (Fig2B and C). Anti-Ly6G treatment was associated with significant impairment of fracture healing. Representative histological section stained with Masson's trichrome at day 14 showed anti-Ly6G treatment led to formation of a large immature and unmineralized callus compared to IgG control treatment (Fig2D). Anti-Ly6G treatment resulted in delayed mineralization and remodeling of the fracture callus at day 28 (Fig2E).

### Addition of rhTNF to the fracture environment promotes the recruitment of neutrophils, monocytes, and CCL2 expression

**Figure 3:**
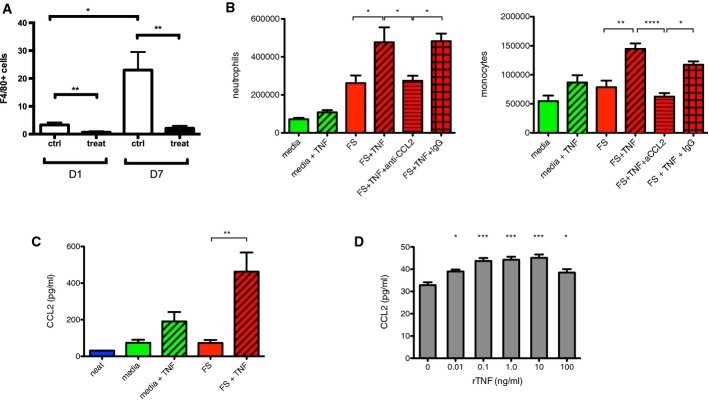
TNF promotes recruitment of innate immune cells and CCL2 expression

Since rhTNF was only effective when given in the first 24 h following injury and the major inflammatory cell infiltrate during this period consisted primarily of TNF-expressing neutrophils, we investigated whether exogenous rhTNF, which led to augmented fracture repair *in vivo*, acted via neutrophils. We hypothesized that additional rhTNF at the fracture site would enhance the innate immune response to promote the recruitment of neutrophils, which in turn attract monocytes that have been shown to be associated with fracture healing (Alexander *et al*, 2011) and orchestrate wound healing in other tissues (Scapini *et al*, 2000; Nathan, 2006; Soehnlein & Lindbom, 2010). We found that anti-Ly6G treatment also depleted the number of neutrophils and F4/80^+^ cells at the fracture site on histology on days 0 and 7 (Figs2B, C and 3A). The analysis of numerous sections at multiple time points and the sacrifice of many animals would be required to translate the static pictures provided by histology into an appreciation of the dynamics of cell migration and chemokine release *in vivo*. Therefore, we used the air pouch model of inflammation, a widely accepted and validated *in vivo* model for studying the regulation of the early events of local inflammation (Romano *et al*, 1997; Lawrence *et al*, 2001), to accurately quantify the neutrophils and monocytes attracted to the complex local fracture cytokine milieu. Media or murine fracture supernatants prepared as described above were injected into the air pouch either alone or in combination with 1 ng rhTNF, and the cellular infiltrates assessed at 4 h. Addition of rhTNF to fracture supernatant into the air pouch resulted in an increase in both the numbers of neutrophils (Ly6G^+^, CD11b^+^ cells) and monocytes/macrophages (Ly6G^−^, CD11b^+^, CD115^+^ cells) (Fig3B and Supplementary Fig S1D).

Anti-Ly6G treatment depletes local monocytes/macrophages at the fracture site. Counts of positively stained infiltrative F4/80^+^ cells in the adjacent muscle to the fracture site following treatment with control or anti-Ly6G at day 1 and day 7. Data are presented as mean ± SEM. **P = 0.005 at day 1 and **P = 0.002 at day 7, and *P = 0.013 for control at day 1 versus control at day 7, by unpaired two-sided *t*-test.

Addition of 1 ng rhTNF to fracture supernatant in the air pouch model led to increased numbers of neutrophils (Ly6G^+^, CD11b^+^ cells) and monocytes/macrophages (Ly6G^−^, CD11b^+^, CD115^+^ cells) in the cellular infiltrate. These effects were abrogated by the addition of anti-CCL2. Neutrophils: FS versus FS + TNF *P = 0.015, FS + TNF versus FS + TNF + anti-CCL2 *P = 0.028, and FS + TNF + anti-CCL2 versus FS + TNF + IgG *P = 0.042 by one-way ANOVA followed by Bonferroni's multiple comparison test. Monocytes/macrophages: FS versus FS + TNF **P = 0.0063, FS + TNF versus FS + TNF + anti-CCL2 ****P < 0.0001, and FS + TNF + anti-CCL2 v FS + TNF + IgG *P = 0.015 by one-way ANOVA followed by Bonferroni's multiple comparisons test.

Addition of 1 ng rhTNF to fracture supernatant increased CCL2 protein levels in the air pouch. “Neat” indicates level of CCL2 in fracture supernatant before injection into air pouch. Data are presented as mean ± SEM. FS versus FS + TNF **P = 0.0020 by one-way ANOVA with Bonferroni's multiple comparisons test.

Addition of rhTNF to enriched bone marrow-derived murine neutrophils pre-exposed to fracture supernatant *in vitro* led to up-regulation of CCL2 production at 1 h. Data are presented as mean ± SEM. No TNF versus TNF 0.01 ng/ml *P = 0.012, TNF 0.1 ng/ml ***P < 0.0001, TNF 1.0 ng ***P = 0.0001, TNF 10 ng/ml ***P < 0.0001, and TNF 100 ng/ml *P = 0.023 by one-way ANOVA with Dunnett's multiple comparisons test.

As monocytes are recruited by chemokines during the inflammatory response (Nathan, 2006; Soehnlein & Lindbom, 2010), we examined the production of the key neutrophil-derived monocyte chemokines CCL2 (MCP-1), CCL3 (MIP-α), and sIL-6R. Addition of 1 ng of rhTNF in the air pouches led to an increase in CCL2 levels as determined by ELISA (Fig3C), while levels of CCL3 and sIL-6R were unaffected (Supplementary Fig S1C). The enhanced recruitment of both neutrophils and F4/80^+^ cells into the air pouch model by TNF was abrogated by the addition of CCL2 neutralizing antibody (Fig3B). Representative FACS plots are shown in Supplementary Fig S1D.

To determine whether rhTNF can promote CCL2 expression by murine neutrophils, we used enriched preparations of murine neutrophils derived from the tibiae of C57BL/6 mice (82.7%) (Supplementary Fig S1E) and incubated them in fracture supernatant and rhTNF at a range of concentrations *in vitro*. Addition of 10 pg or more of rhTNF promoted CCL2 expression in neutrophils at 1 h following exposure to fracture supernatant (Fig3D).

There is controversy in the literature over whether neutrophils are able to produce CCL2. It is possible that the CCL2 production seen in Fig2H may be attributed to contaminant cells other than neutrophils. To determine the ability of murine neutrophils to express CCL2, we used immunocytochemistry to detect co-expression (merged signal: yellow/orange) of neutrophil elastase (green) and CCL2 (red). We collected cells from murine whole blood followed by red cell lysis. Neutrophils were identified by the multi-lobulated morphology of their nuclei on DAPI (blue) and the expression of neutrophil elastase. We then exposed the cells to either control media or fracture supernatant for 30 min followed by 1 ng rhTNF. Murine neutrophils derived from whole blood clearly expressed CCL2 when exposed to a combination of fracture supernatant and TNF, but not to TNF alone (Supplementary Fig S1F).

### CCR2 inhibition impairs fracture repair *in vivo*

The CCL2/CCR2 axis has been found to be important in the healing response of other types of tissue, including skeletal muscle (Weber *et al*, 1999; Surgeons AAoO, 2007; Martinez *et al*, 2010; Lu *et al*, 2011; Arefieva *et al*, 2012; Chan *et al*, 2012a; Chen *et al*, 2012; Liang *et al*, 2012; Suresh *et al*, 2012; Wan *et al*, 2012; Kostarnoy *et al*, 2013). *In vivo* inhibition of CCR2, the G-protein-coupled receptor for CCL2, using small molecule inhibitor INCB3344 as previously described (Brodmerkel *et al*, 2005; Shin *et al*, 2009; Xue *et al*, 2010) significantly impaired endochondral fracture healing compared to vehicle control at day 28 after surgery (Fig1). The micro-CT reconstructions show that CCR2 inhibition led to very poor fracture callus formation, as evidenced by impairment of new bone formation and lack of bridging across the fracture site (Marsh, 1998). However, local administration of rmCCL2 at 10 and 100 ng at the fracture site on days 0 and 1 did not affect fracture repair at day 28 on micro-CT analysis (Supplementary Fig S1G), most likely due to suboptimal pharmacodynamics including the short half-life of the protein and pharmacodynamics (Ruggiero *et al*, 2003).

**Figure 4:**
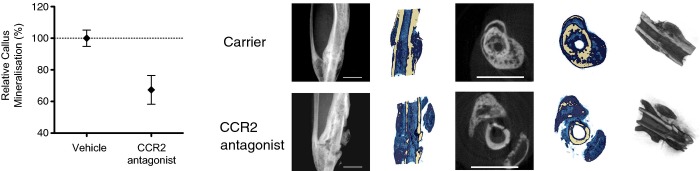
Effects of inhibiting inflammatory cell recruitment on fracture healing Treatment with CCR2 antagonist, INCB3344, led to impaired fracture healing compared to vehicle control as shown by the reduced % callus mineralization at day 28 after operation. Data are presented as mean ± SEM. *P = 0.011 unpaired two-sided *t*-test. Representative micro-CT images are shown. Scale bar, 2 mm.

### Addition of rhTNF augments fracture repair in osteoporotic mice

**Figure 5:**
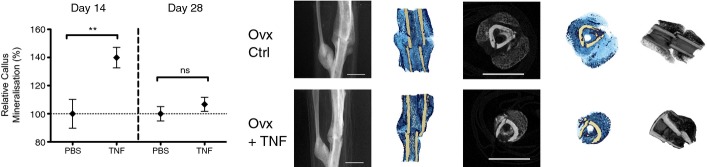
TNF promotes fracture healing in osteoporotic bone rhTNF treatment augmented early phase fracture healing in osteoporotic mice. Treatment with 1 ng rhTNF at the fracture site on days 0 and 1 led to increased % callus mineralization at day 14 but no difference at day 28 indicating accelerated early healing but the same final result at day 28. Data are presented as mean ± SEM. At day 14, PBS control versus TNF, **P = 0.0099; at day 28, PBS control versus TNF, ^ns^*P* = 0.37, by unpaired two-sided *t*-test. Representative micro-CT images at day 14 are shown. rhTNF treatment led to mature callus bridging across the fracture which was absent in the PBS-treated control group. Scale bar, 2 mm.

Osteoporotic fractures represent a huge unmet clinical need. We rendered mice osteoporotic by ovariectomy (He *et al*, 2011) (Supplementary Fig S1H). We then assessed the effect of TNF on fracture healing in these animals. Treatment with 1 ng TNF at the fracture site on days 0 and 1 led to an increased relative callus mineralization at day 14 after surgery, but by day 28, healing was equivalent to PBS-treated controls (Fig5). This indicates that local administration of rhTNF leads to initial accelerated healing to achieve the same final result over a shorter period. The micro-CT images show that rhTNF treatment led to mature callus bridging across the fracture, which was absent in the PBS-treated control group, at day 14.

## Discussion

Fracture repair involves a complex cascade of events involving numerous cell types and the spatially and temporally coordinated release of multiple factors (Dimitriou *et al*, 2005). Studies in humans and mice of the fracture hematoma, which is rich in these factors and immune cells, suggest that the early inflammatory events following fracture are critical to the outcome of fracture healing (Grundnes & Reikeras, 1993; Chung *et al*, 2006; Kolar *et al*, 2010, 2011; Hoff *et al*, 2013). Furthermore, the use of anti-inflammatory or cytotoxic medications, including corticosteroids, non-steroidal anti-inflammatory drugs, or chemotherapeutic agents, during the early stages of fracture healing have been shown to be deleterious (Sudmann *et al*, 1979; Engesaeter *et al*, 1992; Altman *et al*, 1995; Gerstenfeld *et al*, 2003b; Simon & O'Connor, 2007; Dimmen *et al*, 2008; Pountos *et al*, 2008). While these studies support the importance of the early inflammatory events in determining the final outcome of fracture healing, the precise cells and cytokines involved remain unclear.

Inflammation is necessary to initiate the reparative response following injury (Nathan, 2006; Soehnlein & Lindbom, 2010). We have previously found that proinflammatory cytokines, in particular TNF, play an important role in fracture healing through the recruitment and osteogenic differentiation of muscle-derived mesenchymal stromal cells (MSCs) (Glass *et al*, 2011). In the skeletally mature animal, the contribution of the periosteum to fracture healing diminishes as the cambium layer regresses and loses it osteogenic activity (O'Driscoll *et al*, 2001). In our fracture model, the periosteum is stripped to ensure that fracture healing occurs by endochondral healing as seen in the adult human, particularly in high-energy fractures where the periosteum is often stripped and muscle is likely a major contributor of osteoprogenitors (Liu *et al*, 2010; Chan *et al*, 2012b). Others have confirmed that TNF also promotes osteogenic differentiation of bone marrow-derived MSCs in a time- and dose-dependent manner (Mountziaris *et al*, 2010; Lu *et al*, 2012). In the present study, we found that local TNF treatment was only effective in augmenting fracture healing when administered at the fracture site during the first 24 h after surgery (Fig1B). Crucially, this indicates that the early inflammatory phase represents a key rate-controlling step in fracture healing. It is noteworthy and important that this early phase typically coincides with the timing of surgical treatment, which would therefore be a great advantage for potential biologic treatments targeting early inflammation post-fracture. Chronically high levels of TNF are known to impair fracture healing (Alblowi *et al*, 2009). It is interesting to note that the highest dose of TNF in the early inflammatory window did not do so. This may be due to the relatively short stimulation period of osteoclasts. Our current data indicate that rhTNF also plays an important role in augmenting the early inflammatory response. In TNFR-deficient mice, fracture healing was delayed particularly during the early phase (Gerstenfeld *et al*, 2003a). Our gain and loss studies in which exogenous TNF is administered and endogenous TNF expression is depleted in wild-type animals confirm the role of TNF in fracture healing as anti-TNF treatment delayed healing for at least 4 weeks.

Injury activates the innate immune response with recruitment of neutrophils that mount a host defensive response, including phagocytosis and the release of reactive oxygen species, antimicrobial peptides, and serine proteinases (Soehnlein & Lindbom, 2010). Neutrophils are the most abundant leukocyte subset in the blood of humans and mice. While neutrophils have traditionally been regarded as professional phagocytes which clear debris and bacterial pathogens and delay healing, evidence is emerging to support a much wider role in orchestrating downstream events (Weiss, 1989; Nathan, 2006; Bastian *et al*, 2011). Indeed, wounds heal poorly in patients with insufficient neutrophils (Lekstrom-Himes & Gallin, 2000), or patients whose neutrophils are unable to adhere to the endothelium or extracellular matrix (Kong *et al*, 1992; Roos & Law, 2001). While depletion of neutrophils led to a reduction of mesenchymal repair tissue within the injured growth plate cartilage in a rat model (Chung *et al*, 2006), the role of neutrophils in bone healing is currently poorly understood. We found that depletion of neutrophils by anti-Ly6G led to impairment of fracture healing (Fig2D and E) and that this may in part be due to disruption of the downstream events of the inflammatory cascade, including recruitment of monocytes (Fig3A).

Previous studies have reported that neutrophils are able to express TNF and that murine neutrophils contain pre-formed TNF that is rapidly (15 min) mobilized to the cell surface upon cell activation (Bennouna & Denkers, 2005). However, the role of early neutrophil-derived TNF in the inflammatory response is unknown (Bennouna *et al*, 2003; Tsuda *et al*, 2004; van Gisbergen *et al*, 2005). Local expression of TNF is essential for the recruitment of leukocytes to extravascular sites (Tessier *et al*, 1997). *In vitro*, TNF promotes the release of chemokines, including CCL3 (MIP-1α) and sIL-6R, the main neutrophil-derived chemokines responsible for monocyte recruitment (Soehnlein & Lindbom, 2010), as well as CCL2 (MCP-1) (Yamashiro *et al*, 2000). These drive the local recruitment of other inflammatory cells, including monocytes and macrophages (Pliyev, 2008), which are critical for bone repair (Alexander *et al*, 2011). Although compared to monocytes or macrophages, each neutrophil produces lower levels of a given cytokine, neutrophils typically represent the earliest cell type present and greatly outnumber other phagocytes at inflammatory sites, by 1–2 orders of magnitude. Hence, they constitute a very important source of cytokines, including TNF, at a critical juncture of the healing response.

Although CCL2 was originally named “monocyte chemoattractant protein-1” (MCP-1), it has also been shown to be important in the recruitment of neutrophils (Matsukawa *et al*, 1999; Speyer *et al*, 2004) as well as of monocytes and macrophages (Shi & Pamer, 2011), including at the fracture site (Xing *et al*, 2010). CCL2 is produced by a number of cell types including neutrophils (Burn *et al*, 1994; Yoshimura & Takahashi, 2007; Pliyev, 2008; Pelletier *et al*, 2010) and monocytes (Yoshimura *et al*, 1989), as well as endothelial cells (Hu *et al*, 2009), fibroblasts, vascular smooth muscle cells (Xing *et al*, 2007), and bone marrow cells (Cushing *et al*, 1990; Moehle *et al*, 2011). It was detected within the first 24 h following trauma in an *in vitro* fracture hematoma model (Hoff *et al*, 2013). Despite reports that mRNA for CCL2 can been detected in neutrophils (Burn *et al*, 1994; Yamashiro *et al*, 2000), it is unknown whether human or murine neutrophils express the protein. We found that addition of rhTNF to enriched bone marrow-derived murine neutrophils exposed to fracture supernatant *in vitro* led to increased levels of CCL2 by ELISA (Fig3C). Notably, we were also able to directly visualize murine neutrophils derived from bone marrow readily expressing CCL2 protein by immunocytochemistry when they were exposed to fracture supernatant and rhTNF but not rhTNF alone (Supplementary Fig S1F). Our findings add to a previous report which demonstrated that TNF upregulated CCL2 mRNA by human neutrophils *in vitro* when co-stimulated with TLR ligands (Yamashiro *et al*, 2000). Our finding that both neutrophil depletion and CCR2 antagonism lead to impaired fracture healing supports the crucial role of neutrophil-derived CCL2 in fracture repair (Figs2D, E and 4).

Our observation that addition of TNF led to enhanced monocyte/macrophage recruitment is notable as this cellular subset has been implicated in osteogenesis (Guihard *et al*, 2012; Nicolaidou *et al*, 2012) and fracture callus remodeling (Alexander *et al*, 2011). Recently, a population of macrophage-like cells, or “osteomacs”, that line the bone surface osteoblasts have been described in both mice and humans. These cells promote osteoblast mineralization *in vitro* and are critical in the maintenance of mature osteoblasts *in vivo* (Chang *et al*, 2008) as well as bone formation in a murine model of bone injury (Alexander *et al*, 2011). Depletion of “osteomacs” led to concurrent reduction in the number of osteoblasts. Although “osteomacs” are derived from the myeloid lineage, they are distinct from multinucleated osteoclasts and it is likely that their precursor cells diverge from a common progenitor earlier in the myeloid lineage and mature along independent pathways to perform different functional roles. “Osteomacs” therefore contribute to the regulation of bone formation that is unrelated to osteoclastic bone resorptive activity. However, how the prevailing cytokine environment affects the osteogenic activity of “osteomacs” remains to be elucidated.

We previously found that exposure to rhTNF enhanced CCL2-mediated recruitment of osteogenic precursor cells (Glass *et al*, 2011). Up-regulation of the CCL2/CCR2 axis has also been shown to promote healing in various types of tissue, including skeletal muscle, lung, and endothelium as well as bone through the recruitment of monocytes/macrophages and MSCs (Weber *et al*, 1999; Martinez *et al*, 2010; Lu *et al*, 2011; Arefieva *et al*, 2012; Chan *et al*, 2012a; Chen *et al*, 2012; Liang *et al*, 2012; Suresh *et al*, 2012; Wan *et al*, 2012; Kostarnoy *et al*, 2013). CCR2-deficient mice exhibited impaired healing in a closed fracture model (Xing *et al*, 2010). However, genetically modified mice such as the osteopontin-deficient mice often display subtle skeletal phenotypes due to functional redundancy in gene family members (Davey *et al*, 2004) that may influence the interpretation of data on complex physiological processes such as fracture healing. Our finding that CCR2 inhibition using a small molecule inhibitor impairs healing confirms the importance of the CCL2/CCR2 axis (Fig4). It is notable that CCR2 inhibition led to the greatest impairment of healing in our fracture model, and showed a tendency toward non-union, a complication that particularly affects open tibial fractures in patients. Non-union is a failure of fracture repair and is characterized by the absence of fracture callus formation with no bridging across the fracture gap (Marsh, 1998). Clinically, this typically requires further operative procedures to stimulate healing including excision of the intervening fibrous tissue and juxtaposition of the surgically “freshened” bone ends, which may in effect serve to “reset” the biological clock through a controlled inflammatory insult. INCB3344 has been shown to be a highly selective (> 100-fold) inhibitor for CCR2 compared to the closest related chemokine receptor subtypes, including CCR1 and CCR5, and hence has limited off-target effects (Xue *et al*, 2010). CCR2 has been shown to be crucial in the recruitment of macrophages to sites of injury, and the movement of monocytes from the bone marrow into the bloodstream and site of traumatic injury (Kuziel *et al*, 1997; Ma *et al*, 2002; Schober *et al*, 2004; Tsou *et al*, 2007). However, monocytes and macrophages also contribute to other aspects of fracture healing including the expression of osteogenic cytokines such as TNF and IL-6 (Yang *et al*, 2007; Mountziaris *et al*, 2010; Glass *et al*, 2011; Lu *et al*, 2012), and osteoinductive growth factors such as BMP-2 (Champagne *et al*, 2002) as well as osteoprogenitor differentiation, osteoclast differentiation, and vasculogenesis (Xing *et al*, 2010). Therefore, it is possible that the drastic impairment of fracture repair is a consequence of the disruption of multiple CCR2-dependent processes. In our model, the local addition of rmCCL2 did not augment fracture repair (Fig1G), but this may be due to a number of reasons, including the short half-life of the protein and low bioavailability of the protein (Ruggiero *et al*, 2003). Strategies to prolong the half-life of CCL2 including PEGylation, fusion proteins, antibody complexes, and mutagenesis may be utilized to further test the therapeutic potential of up-regulating CCL2.

Osteoporosis is characterized by low bone mass and weakened bone structure, leading to 1.5 million fragility fractures in the USA every year (Holroyd *et al*, 2008). Half of all patients who sustain fragility fractures involving the femoral neck are permanently disabled, and the mortality rate is 21–36% within the first year (Eisman *et al*, 2012). Hence, there remains a pressing need to develop effective strategies to accelerate healing of fragility fractures. To this end, we examined whether TNF treatment would also be effective in mice that have been rendered osteoporotic by oophorectomy. We found a 40% improvement in healing at 2 weeks and equivalent mineralization to PBS controls at 4 weeks (Fig5). Rates of recovery and mobilization in patients with fragility fractures are critically dependent on fracture healing as premature loading leads to implant failure, accounting for the excessive morbidity and mortality seen in this vulnerable group of patients. Therefore, acceleration of healing during the early phase of fracture repair is particularly relevant in the clinical setting.

**Figure 6:**
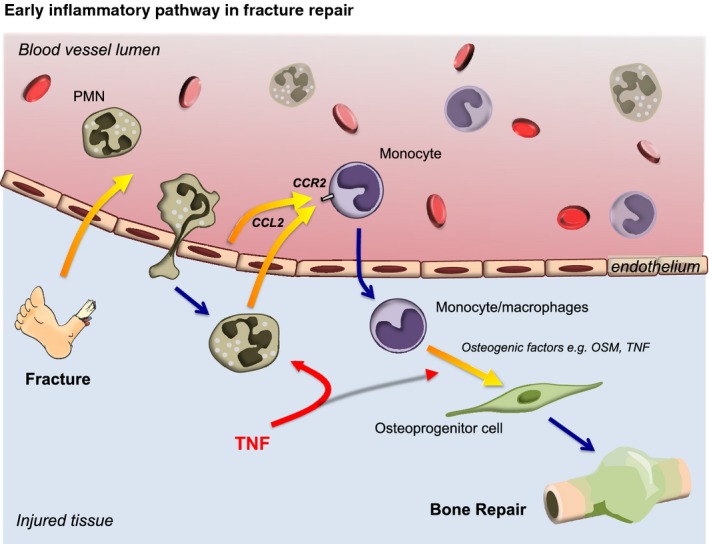
Definition of the early fracture healing pathway Schematic detailing the role of the early inflammatory pathway in fracture healing. Addition of local rhTNF promotes the recruitment of neutrophils and monocytes via up-regulation of CCL2, leading to augmentation of fracture repair.

In summary, our combined *in vivo* and *in vitro* data show that addition of low-dose rhTNF during the early inflammatory response promotes the recruitment of neutrophils and monocytes via up-regulation of CCL2 and leads to acceleration of fracture repair. Our data show that the role of neutrophils is not simply limited to clearance of pathogens and cellular debris. They also orchestrate the next stage of resolution and regeneration through the recruitment of monocytes. Mechanistically our data suggest that local administration of a low dose of recombinant TNF at the fracture site shortly after injury acts via two mechanisms. Firstly, it potentiates and augments the early innate immune response comprising neutrophils followed by monocyte/macrophage recruitment to promote the physiological healing processes through the CCL2/CCR2 axis (Fig6). Secondly, it promotes the recruitment and osteogenic differentiation of MSCs (Glass *et al*, 2011).

This study has a number of limitations. First, we used percentage callus mineralization as the main parameter of fracture repair. While biomechanical testing is considered as an alternative gold standard in the assessment of bone quality, this is most reliable in larger bones, such as femora of rats. Bend testing is used routinely for intact bones, but we have found bend testing to be inconsistent when testing the effects of biological therapies on fractured mouse tibiae. Indeed, analysis of micro-CT parameters has been shown to be a more sensitive method of evaluating murine fracture callus properties than biomechanical studies (O'Neill *et al*, 2012). The total and mineralized callus volumes from which the percentage callus mineralization values were derived are shown in Supplementary Fig S2. Second, while our data show either augmentation or impairment of fracture healing at either day 14 or day 28 post-fracture, it is not possible to comment on whether the changes observed are due to a change in the rate or quality of fracture repair, or indeed both. This is because the mice were sacrificed at each time point. For future investigation, *in vivo* micro-CT imaging would enable us to follow the progression of fracture repair longitudinally over time, with the advantage that each mouse will serve as its own control to allow matched analyses.

By systematically unraveling the initial events in the fracture-healing pathway, we have identified the potential of enhancing the early innate immune response following fracture to augment fracture repair. This has profound implications in the clinical setting. For example, during surgical treatment of fractures, especially open fractures, surgeons often lavage the wound extensively in order to reduce the risk of infection, but also unintentionally deplete the wound bed of crucial mediators as well as immune and osteoprogenitor cells. Therapeutic up-regulation of the innate immune system may be especially relevant under these circumstances. However, of greatest potential clinical significance is the efficacy of this novel regenerative therapy in osteoporotic bone, the major unmet clinical need.

## Materials and Methods

### Murine fracture model

Animal fracture model procedures were approved by the institutional ethics committee and the United Kingdom Home Office (PLL 71/7161). Skeletally mature (12–14 week old) female C57/BL6 mice were obtained from Charles River, UK. Normal chow and drinking water were provided *ad libitum*. The murine fracture procedure was performed as previously described (Harry *et al*, 2008). Briefly, an incision is made over the tibia. The periosteum is stripped circumferentially, the medullary canal is reamed, and a 0.38-mm intramedullary fixation pin is inserted. An osteotomy is created at the junction of the middle and distal thirds of the tibia and the fixation pin cut flush with the tibial cortex and skin closed directly with non-absorbable sutures.

Anti-TNF: TN3-19.12, a neutralizing hamster IgG1 anti-TNF-α/β mAb (Leinco Technologies) or IgG control was given (1 mg) via intraperitoneal injections on days 0, 2, 5, and 8 after operation.

rmIL10 (R&D Systems) was injected locally at the fractures site at 10 ng on days 0 and 1; PBS was used as vehicle control.

Anti-IL10R or IgG isotype control (R&D Systems) was given (2 μg) via intraperitoneal injections on day 1 before operation and days 0, 1, 4, 6, and 8 after operation.

Anti-Ly6G-specific monoclonal antibody, 1A8, or IgG_2A_ isotype control, 2A3 (BioXCell) (Daley *et al*, 2008), was given (0.5 mg) via intraperitoneal injections on days 1 and 3 before and after operation.

The CCR2 antagonist, INCB3344 (Brodmerkel *et al*, 2005; Shin *et al*, 2009; Xue *et al*, 2010; Xie *et al*, 2011; Chan *et al*, 2012a), was administered at 30 mg/kg/day via daily intraperitoneal injections starting 3 h before operation and continuing until and including day 8 after operation (Haoyuan Chemexpress Co Ltd). Vehicle control was 10% DMSO/0.9% carboxymethylcellulose.

rmCCL2 (R&D Systems) was injected locally at the fracture site 10 ng or 100 ng on day 0 and day 1; PBS was used as vehicle control.

Fig1A: TNF 0 ng (*n* = 8), 0.01 ng (*n* = 7), 1 ng (*n* = 6), 100 ng (*n* = 8)
Fig1B: Ctrl (*n* = 9), D0 + 1 (*n* = 6), D0 (*n* = 10), D1 (*n* = 7), D2 (*n* = 8), D3 (*n* = 6)
Fig1C: Ctrl (*n* = 10), anti-TNF (*n* = 6), rmIL-10 (*n* = 8)
Fig2A: Ctrl (*n* = 6), 0.25 (*n* = 6), 0.5 (*n* = 12), 1 (*n* = 9), 3 (*n* = 10), 6 (*n* = 6), 12 (*n* = 6), 24 (*n* = 10), 72 (*n* = 6)
Fig2B (left): IgG ctrl (*n* = 8), Anti-Ly6G (*n* = 9); (right) *n* = 3 per group
Fig2E: IgG Ctrl (*n* = 8), Anti-Ly6G (*n* = 9)
Fig3A: (*n* = 3) per group
Fig3B: media (*n* = 11) media + TNF (*n* = 12), FS (*n* = 8), FS + TNF (*n* = 11), FS + TNF + anti-CCL2 (*n* = 8), FS + TNF + IgG (*n* = 8)
Fig3C: media (*n* = 6), media + TNF (*n* = 6), FS (*n* = 6), FS + TNF (*n* = 6)
Fig3D: *n* = 3 per group
Fig4: vehicle (*n* = 6), CCR2 antagonist (*n* = 7)
Fig5: Day 14 PBS (*n* = 6), TNF (*n* = 6), Day 28 PBS (*n* = 7), TNF (*n* = 7)

### Micro-CT scanning and analysis

Mouse tibiae were harvested at 14 or 28 days post-fracture and fixed in 70% ethanol. Each bone was scanned with a Skyscan 1174 scanner (SkyScan, Kontich, Belgium), 50 kV, 800 μA, and 8.3 μm isometric voxel resolution, and 0.7 degree rotation step. Bones exhibiting angulation, rotation, or hypertrophic non-union were excluded. Scans were analyzed using SkyScan CTAnalyser software, version 1.9.3.0 (SkyScan, Kontich, Belgium). Each bone was analyzed by selecting a volume of interest commencing at the fracture site and proceeding proximally for 363 slices (3 mm in height). This region was selected to avoid potential confounding effects of the fibula. Total callus volume was delineated from surrounding tissues and the original bone of the tibia using hand-contoured regions of interest. Mineralized callus volume was determined using global thresholding at a density range calibrated to bone mineral density of 350 gm/cm^3^, as previously described (Harry *et al*, 2008). Percent callus mineralization was calculated as mineralized callus volume divided by total callus volume and normalized against vehicle (PBS) or IgG isotype control.

### *In situ* hybridization

Murine lower limbs were fixed in 4% paraformaldehyde overnight and decalcified for 6 weeks in a 50% mixture of 20% EDTA and 4% paraformaldehyde. Care was taken during processing and section cutting to avoid RNase contamination.

Specific localization of *Tnf* mRNA was accomplished by *in situ* hybridization using an antisense riboprobe synthesized with T7 RNA polymerase using ^35^S-UTP (~800 Ci/mmol; Perkin Elmer UK) and IMAGE clone 40126376 (Source BioScience UK) containing the sequence of interest. A clone was grown in the presence of kanamycin, purified using a Qiagen maxi prep kit, and the resultant plasmid linearized with SpeI to prepare a template that made a 768 base antisense riboprobe that was used without hydrolysis. The region of sequence used to produce the riboprobe did not show significant homology to any other known mouse mRNA sequences in the RefSeq.

The methods for pre-treatment, hybridization, washing, and dipping of slides in Ilford K5 for autoradiography were performed as previously described (Steddon & Cunningham, 2005) for formalin-fixed paraffin-embedded tissue with some modifications (Fromigue *et al*, 2009).

The presence of hybridizable mRNA in all compartments of the tissues studied was established in near serial sections using an antisense β-actin probe. Autoradiography was carried out at 4°C (two exposures per section at 8 and 15 days) before developing in Kodak D19 and counterstaining by Giemsa's method. Sections were examined under conventional or reflected light dark-field conditions (Nikon Eclipse ME600 epi-illumination microscope with Q imaging MicroPublisher 5.0 camera) that allowed individual autoradiographic silver grains to be seen as bright objects on a dark background.

### Immunohistochemistry

Monocytes/macrophages: Limbs were fixed in 4% paraformaldehyde overnight and decalcified for 6 weeks in 15% EDTA. The limbs were bisected longitudinally and paraffin-embedded, and 4-μm sections were cut. Immunohistochemistry (IHC) was performed on deparaffinized and rehydrated sections, with specific primary antibody against F4/80 (rat anti-mouse (Biolegends)), as previously described (Alexander *et al*, 2011), or anti-neutrophil elastase (Abcam). All sections were counterstained using Mayer's haematoxylin (Leica Biosystems, UK). Tissue staining was viewed using an Olympus BX51 microscope with an Olympus DP71 camera and DP Manager (Olympus) imaging software.

### Quantification of circulating neutrophils

Mice were anaesthetized with pentobarbital until unresponsive to stimuli, and a cardiac puncture was performed. Blood was collected into a syringe containing 100 μl sodium citrate. Blood smears were stained with DiffQuik for the enumeration of neutrophils and mononuclear cells. Six animals were used per time point.

### Murine fracture supernatant

C57BL/6 mice were anaesthetized, and closed fractures were created in the lower limbs. Mice are culled at 3 h post-operatively, and the fractured bone segments are harvested and incubated in serum-free DMEM + 1% penicillin/streptomycin for 16 h. Cytokine levels were measured using a pro-inflammatory 7-plex plate and a SECTOR Imager 2400 reader (Mesoscale Discovery).

### Air pouch experiments

Ten- to 12-week C57BL/6 female mice (Charles River, UK) were used. Air pouch model procedures were approved by the institutional ethics committee and the United Kingdom Home Office (PLL 70/7335). Air pouches were produced as previously described (Vestergaard *et al*, 2005; Wilting *et al*, 2007; Mosekilde *et al*, 2011) on the dorsum of the mice by subcutaneous injection of 5 ml sterile air on day 0 and 2 ml on day 4. On day 5, the animals were randomized into groups (minimum six animals per group) and the experiment was conducted in a double-blinded manner. Air pouches were injected with 400 μl of media, fracture supernatant, or fracture supernatant and 1 ng of rhTNF. The animals were sacrificed, and air pouches lavaged with 2 ml of PBS at 4 h post-injection. The exudate was centrifuged at 1,500 for 5 min, and pellets containing the migrated cells were re-suspended in fresh PBS for counting using a hematocytometer following red cell lysis treatment. Characterization of leukocyte subpopulations migrating into the pouch space was determined by flow-cytometry staining with Ly-6G PE (clone 1A8, Miltenyi Biotec, UK), CD11b FITC (clone M1/70, eBioscience, UK) and CD115 APC (clone AFS98, eBioscience, UK) antibodies. PMNs were identified as Ly-6G^+^, CD11b^+^ cells, while the monocytic cells were identified as Ly-6G^−^, CD11b^+^, and CD115^+^ cells as described previously (Vestergaard *et al*, 2005). The levels of mCCL2 cytokine in the exudates were analyzed with a SECTOR Imager 2400 reader (Mesoscale Discovery).

### *In vitro* neutrophil experiment

Neutrophils are enriched to 82.7% purity (Supplementary Fig S1E) using a neutrophil isolation kit according to manufacturer's instructions (Stem Cell Technology) and incubated in 180 μl murine fracture supernatant + 10% FCS + 1% penicillin/streptomycin at 37°C, 4% CO_2_, and a density of 275,000 cells per well in a 96-well plate. rhTNF or PBS (in 20 μl) is added at 30 min. Cells and supernatants are harvested at 1 h. Protein levels of mCCL2 cytokine in the supernatants were analyzed as described above. Experiments were performed in triplicates.

### Immunocytochemistry

Bone marrow cells are extracted from murine tibiae under sterile conditions. The cells are washed, centrifuged, and suspended at 200,000 cells per ml. They were plated on glass coverslips and then exposed to either media + rhTNF at 1 ng/ml (control) or fracture supernatant + rhTNF at 1 ng/ml (treatment). After 4 h, cells were fixed with 4% paraformaldehyde in PBS for 15 min at 37°C, quenched with 50 mM NH_4_Cl/PBS for 10 min, and permeabilized with 0.1% Triton X-100 in PBS for 5 min. Samples were blocked with 3% BSA in PBS for 30 min at room temperature followed by incubation with rabbit anti-neutrophil elastase and rat anti-CCL2 diluted in 3% BSA in PBS for 1 h at room temperature. After washing steps, staining with the appropriate secondary antibody was performed. Neutrophil elastase staining was performed with Alexa Fluor 488, CCL2 was stained with Alexa Fluor 594, and the nucleus was stained with DAPI. Samples were mounted with ProGold antifade mounting media and imaged with a fluorescence microscope.

**The paper explained**

**Problem**

Bone factures are very common. While the majority heal uneventfully, certain sub-groups would benefit from strategies to accelerate healing. These include high-energy open fractures, which are limb-threatening injuries prone to delayed and non-union, and fragility fractures in osteoporotic bone. The rates of recovery and mobilization are limited by the fracture healing time in patients with fragility fractures, thus accounting for the excessive morbidity and mortality seen in this vulnerable group of patients. There is currently no approved therapy for accelerating healing of fragility fractures.

**Results**

Using a combined *in vivo* and *in vitro* approach, we demonstrate the importance of the early innate inflammatory response in the healing of bone fractures. Local administration of TNF, a pro-inflammatory cytokine, within 24 h of injury accelerates fracture repair *in vivo*. Conversely, administration of either anti-TNF or rm-IL-10, an anti-inflammatory cytokine, impairs fracture healing. TNF is expressed by both neutrophils and monocytes/macrophages following fracture. Addition of recombinant TNF to the fracture environment up-regulates the innate immune response by promoting the recruitment of neutrophils followed by monocyte/macrophages, the latter through the up-regulation of the monocyte chemoattractant, CCL2. Depletion of neutrophils impairs fracture repair *in vivo*. While the production of CCL2 by neutrophils is controversial in the literature, we demonstrate enhanced expression of CCL2 in neutrophils exposed to fracture environment following addition of TNF using immunocytochemistry. Macrophages are known to play a key role in orchestrating endochondral bone repair. Inhibition of CCR2, the main receptor for CCL2, found on monocytes/macrophages, led to significant impairment of fracture repair *in vivo*. These data demonstrate the role of the innate immune response via the TNF/CCL2/CCR2 axis in fracture repair.

**Impact**

This is the first direct evidence on the importance of the innate immune response in fracture healing and how it may be targeted to accelerate fracture repair. Our observation that this strategy pertains to osteoporotic bone *in vivo* is of particular interest as clinical translation would address one of the major unmet medical needs of the 21^st^ century.

### Statistical analysis

Statistical analyses were performed using GraphPad Prism version 6 (GraphPad software, San Diego, CA). For every figure, statistical tests are justified as appropriate. All data were expressed as mean ± standard error of the mean (SEM). The significance of the difference between two groups was determined with a one- or two-sided unpaired *t*-tests. Multiple comparisons were made using one-way analyses of variance (ANOVA) followed by Bonferroni or Dunnett post-test analysis for multiple groups. Exact *P*-values are provided for *t*-tests, and multiplicity-adjusted *P*-values are provided for multiple comparisons. *P *< 0.05 are considered statistically significant. Significant results are expressed using asterisks, where *P < 0.05, **P < 0.01, ***P < 0.001, and ****P < 0.0001.

## Supporting Information

Supplementary Figure Legends

Review Process File
