# Supplementary figures and images for: Low-dose TNF augments fracture healing in normal and osteoporotic bone by up-regulating the innate immune response
Source: EMBO Mol Med. 2015 Mar 14;7(5):547–61. doi: 10.15252/emmm.201404487 (PMC4492816; doi:10.15252/emmm.201404487)

# Supplemental Figure 1

**a**

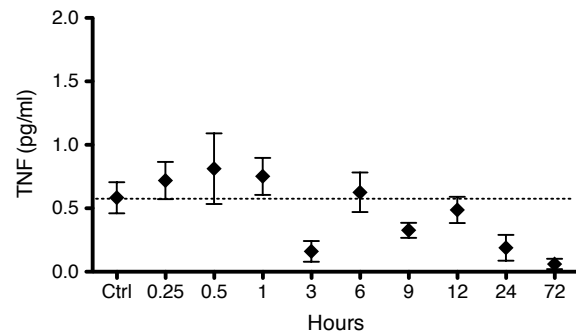

**b**

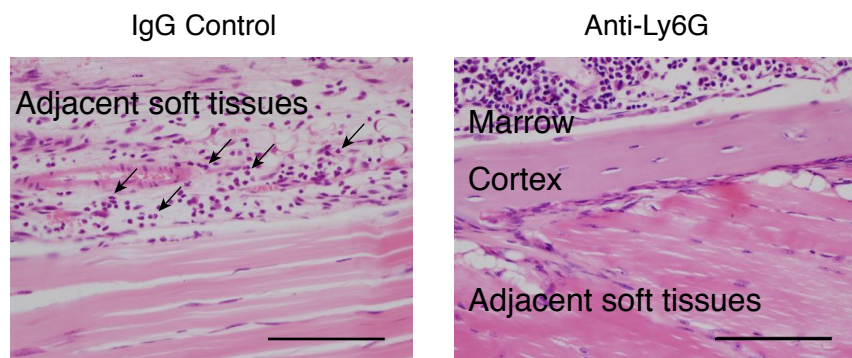

**c**

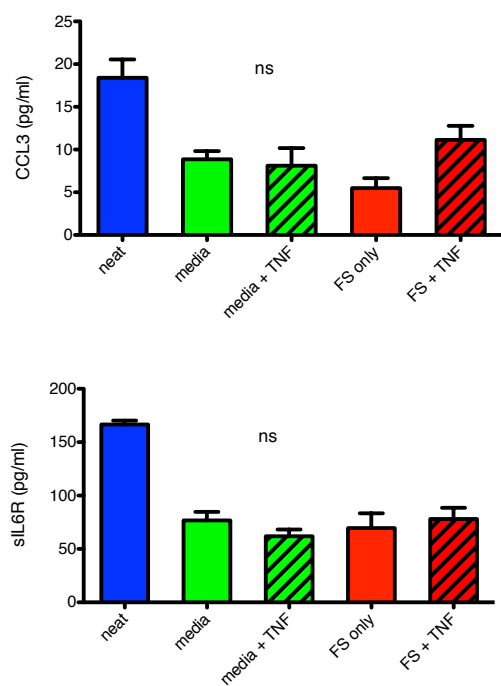

**d**

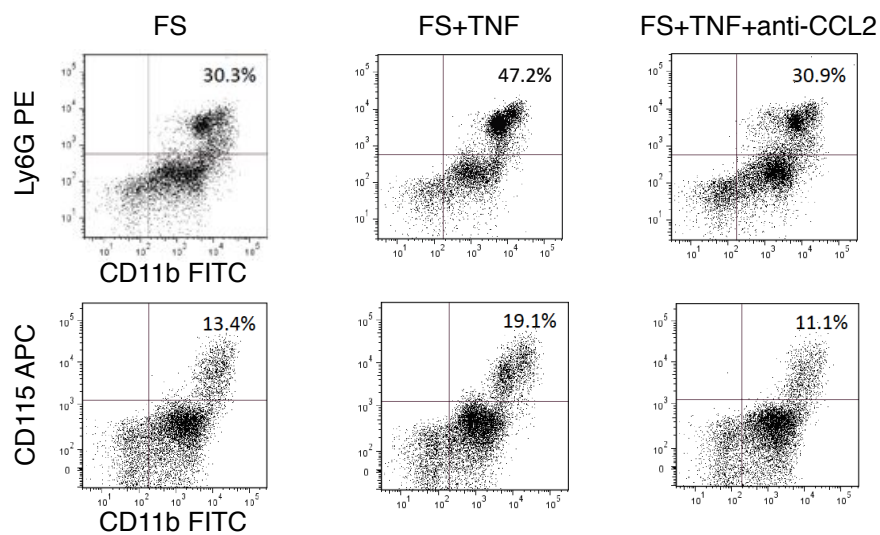

**e**

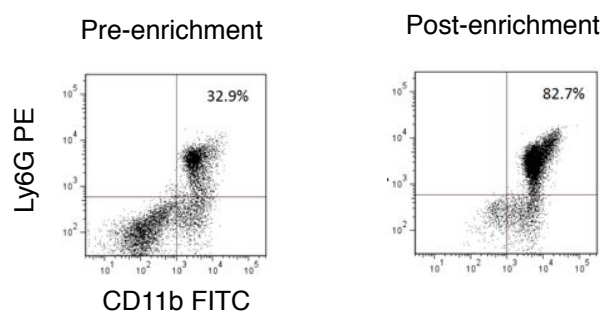

**f**

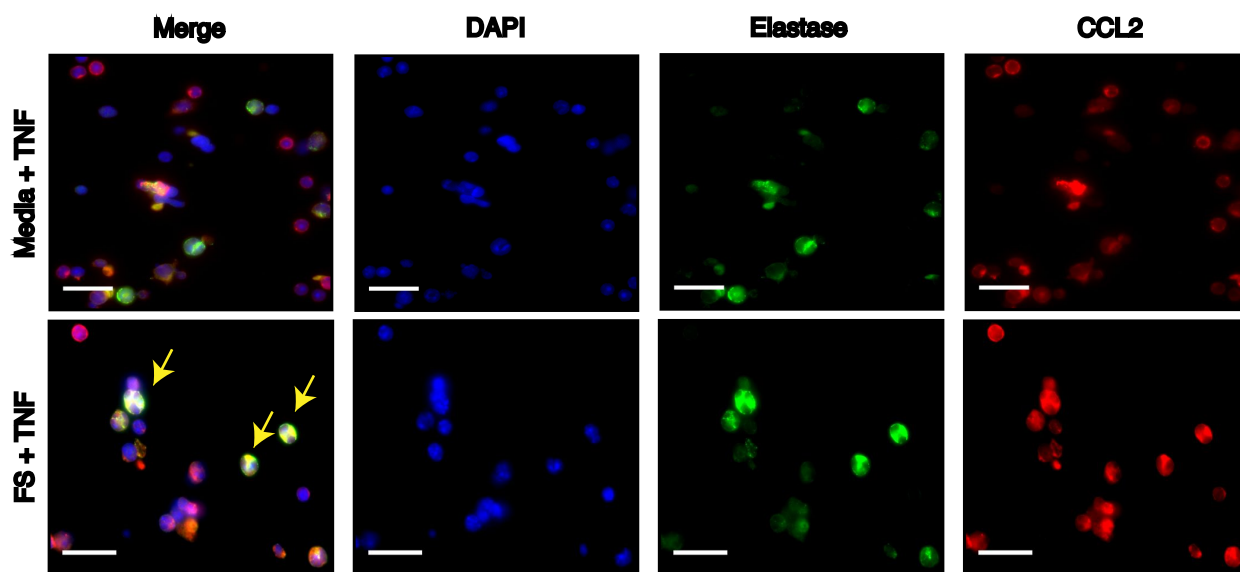

**g**

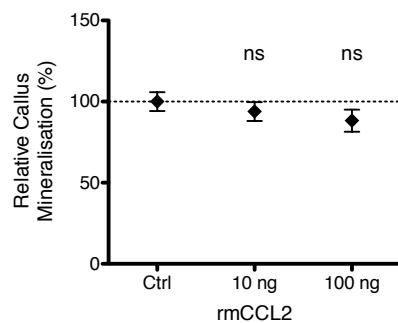

**h**

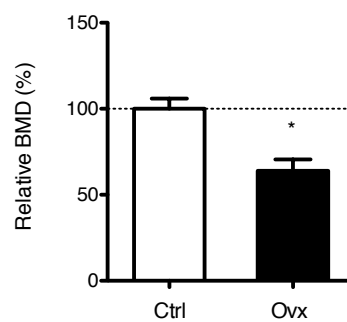

Supplement: Supplementary file 1 [file emmm0007-0547-sd1.pdf]

Supplemental Figure 2

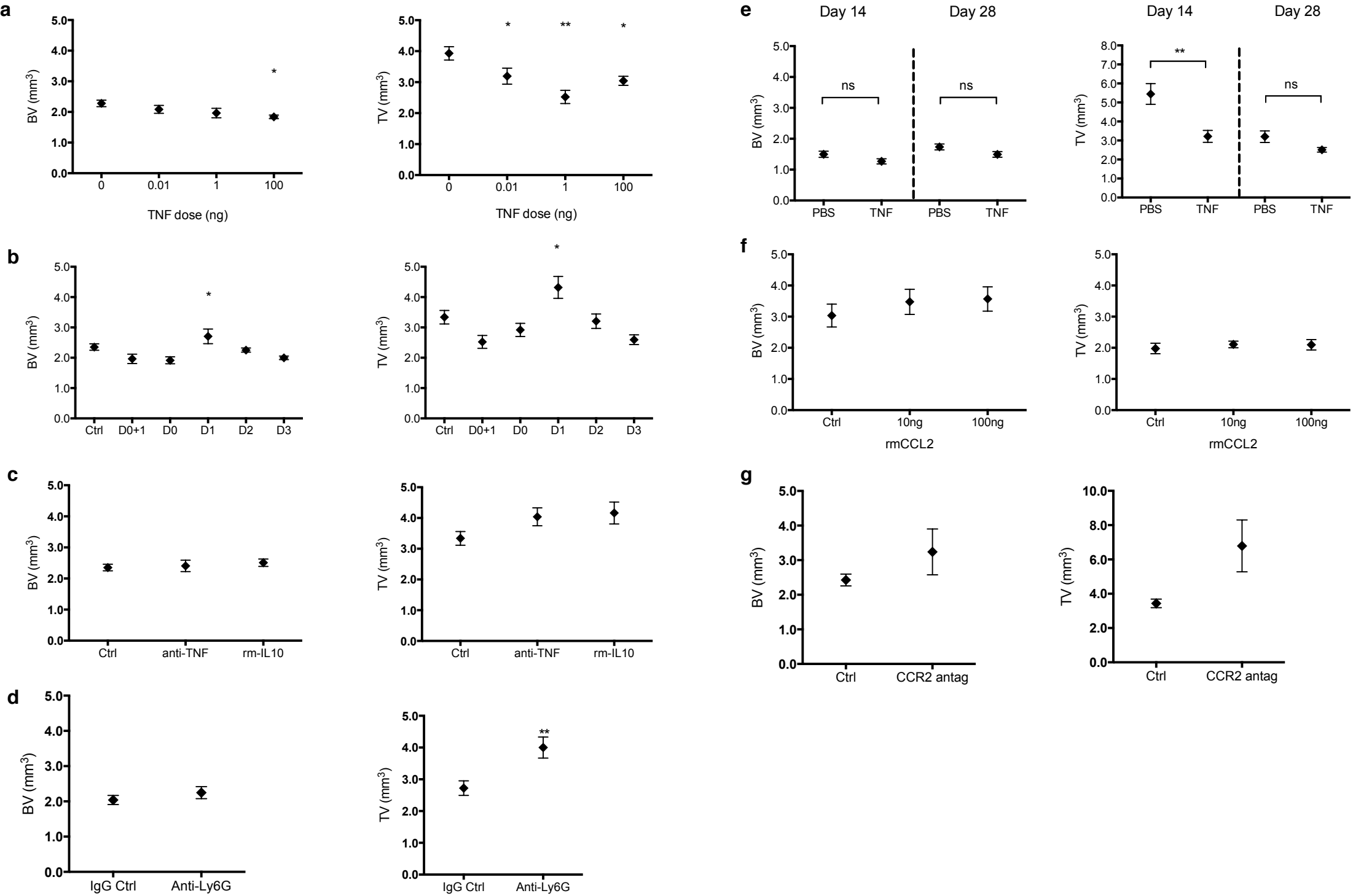

Supplement: Supplementary file 2 [file emmm0007-0547-sd2.pdf]
