# Supplementary material for: Low-dose TNF augments fracture healing in normal and osteoporotic bone by up-regulating the innate immune response
Source: EMBO Mol Med. 2015 Mar 14;7(5):547–61. doi: 10.15252/emmm.201404487 (PMC4492816; doi:10.15252/emmm.201404487)
Supplement: Supplementary file 3 [file emmm0007-0547-sd3.docx]

**Supplementary Figure Legends**

Suppl Figure 1:

a: Time course of circulating serum TNF levels from 15 minutes to 72 hours following tibial fracture. Control represents circulating serum TNF levels in mice without fracture.

b: Representative H&E images showing neutrophil depletion by systemic anti-Ly6G treatment at the fracture site. Neutrophils can be identified by their polymorphonuclear appearance in the adjacent soft tissues at the fracture site (examples indicated by arrows) in the IgG control group but not in the anti-Ly6G treatment group.

c: Addition of 1 ng of rhTNF to fracture supernatant did not affect CCL3 or sIL-6R

in the air pouch (n=6 per group). ‘Neat’ indicates level of CCL3 or sIL-6R in fracture supernatant before injection into air pouch. Data are presented as mean + SEM. No significance on 1-way ANOVA with Bonferroni’s multiple comparisons test.

d: rhTNF-induced promotion of neutrophils influx is abolished by addition of neutralizing antibody to CCL-2. Representative FACS plots with percentage of cells that are Ly6+, CD11b+ (neutrophils) (top row), and Ly6G-, CD11b+, CD115+ cells (monocytes/macrophages) (bottom row).

e: Purity of Ly6+, CD11b+ cells (neutrophils) in percentage using an immunomagnetic negative murine neutrophil enrichment kit (Stem Cell Technology).

f: Using immunocytochemistry, CCL2 expression was detected in neutrophils pre-exposed to fracture supernatant and TNF (examples indicated by yellow arrows). The presence of CCL2 immunoreactivity is seen by red fluorescent and co-localization with neutrophil elastase expression (green) led to a yellow signal in the merged images. The polymorphonuclear morphology of neutrophils was observed in the DAPI (blue) channel.

g: Local addition of rmCCL2 at the fracture site immediate after the operation and again at 24 hours did not affect fracture healing in vivo, as indicated by % callus mineralization (n=6 per treatment group). Data are presented as mean + SEM. No significant changes detected using 1-way ANOVA with Dunnett’s multiple comparisons test.

h: Ovariectomy induces osteoporosis by 4 weeks post-surgery (n=10 per treatment group). BMD was measured by microCT analysis of tibiae of C57BL6 mice. *p=0.0, by unpaired 2-sided t-test.

Suppl Figure 2:

Bone volume (BV) and total callus volume (TV) are presented here in addition to the % callus mineralization data already presented in the main figures for the different treatment groups. Data are represented as mean + SEM. *p<0.05, **p<0.01 by 1-way ANOVA using Dunnett’s multiple comparisons test when >3 groups, or unpaired 2-way t-test. *n* numbers are as per indicated in the main figure legends.

1. Dose response of rhTNF added locally at the fracture site
2. Addition of rhTNF at the fracture site given at different times post fracture
3. Treatment with systemic anti-TNF or local rmIL-10
4. Neutrophil depletion using systemic anti-Ly6G on fracture repair
5. Local TNF treatment on fracture repair in osteoporotic mice
6. Local addition of CCL2 on fracture repair
7. Inhibition of CCR2 using CCR2 antagonist, INCB3344, on fracture repair.
