# Supplementary material for: Low-dose TNF augments fracture healing in normal and osteoporotic bone by up-regulating the innate immune response
Source: EMBO Mol Med. 2015 Mar 14;7(5):547–61. doi: 10.15252/emmm.201404487 (PMC4492816; doi:10.15252/emmm.201404487)
Supplement: Supplementary file 4 [file emmm0007-0547-sd4.pdf]

Low-dose TNF augments fracture healing in normal and osteoporotic bone by up-regulating the innate immune response

James K. Chan, Graeme E. Glass, Adel Ersek, Andrew Freidin, Garry A. Williams, Kate Gowers, Ana I. Espirito Santo, Rosemary Jeffery, William R. Otto, Richard Poulsom, Marc Feldmann, Sara M. Rankin, Nicole J. Horwood, Jagdeep Nanchahal

Corresponding author: Jagdeep Nanchahal, University of Oxford

Review timeline:

Submission date:	23 September 2013
Editorial Decision:	18 October 2013
Resubmission :	18 August 2014
Editorial Decision:	19 September 2014
Revision received:	07 December 2014
Editorial Decision:	12 January 2015
Revision received:	01 February 2015
Accepted:	13 February 2015

Transaction Report:

(Note: With the exception of the correction of typographical or spelling errors that could be a source of ambiguity, letters and reports are not edited. The original formatting of letters and referee reports may not be reflected in this compilation.)

Editor: Céline Carret

1st Editorial Decision 18 October 2013

Thank you for the submission of your manuscript "Low-dose TNF accelerates fracture healing by up-regulating the innate immune response." We have now heard back from the two referees whom we asked to evaluate your manuscript. While they find the study potentially interesting, they have raised too many issues that preclude publication at this stage.

As you will see from the comments below, the referees' reports are very consistent in that they both acknowledge the interest and potential clinical relevance of the findings. Unfortunately, they also highlight a number of serious conceptual and experimental shortcomings of the study, and feel that, given these limitations, your conclusions appear to not be fully supported by the data.

As clear and conclusive insights into a novel clinically relevant observation is key for publication in EMBO Molecular Medicine, and together with the fact that we only accept papers that receive enthusiastic support upon initial review, I am afraid that I cannot see other choice than to return the manuscript to you with the message that we cannot offer to publish it.

I wish to add that, considering the interesting nature of these findings, we would have no objection to evaluate a new manuscript on the same topic if at some time in the near future you would have

obtained data to considerably strengthen the message of the study and address the Reviewers' concerns. Please consider, however, that if you were to send a new manuscript this would be treated as a new submission rather than a revision and would be reviewed anew.

I am sorry to disappoint you on this occasion. I hope, however, that the Reviewers' comments will be helpful for your continued work in this area.

***** Reviewer's comments *****

Referee #1 (Comments on Novelty/Model System):

Technical quality: I rated this medium, because the majority outcome parameter to assess fracture healing is % callus mineralization. While this is a good measure, the analysis is not thorough. The gold standard includes bone and cartilage histomorphometry as well as biomechanical outcomes. I would not reject a paper without these additional data, but the authors are encouraged to be more thorough. They make comparisons to other research that are not in complete agreement with their data, and the authors attribute the differences to the fact that the previous research was performed in GM animals while there work was with small molecule inhibitors. Their explanation does not make much sense to me as it is written.

Novelty: I rated this medium, because the main healing outcomes have been published previously (2011). The new data here is on the CCL2/CCR2 axis and macrophage involvement after TNFa treatment. I think this is more than an incremental advance, but this could have been included in the previous paper.

Medical Impact: I rated this high, because if the authors are correct a new treatment modality may be feasible.

Referee #1 (Remarks):

This is a very interesting paper that describes the effect of TNFa on fracture healing, and indicates that the function of TNFa is mediated by influx of macrophages into the fracture site. Overall, this is an interesting story that could have important clinical impact. The data are restricted to analysis of % mineralization of the fracture callus, with no tissue level (bone vs. cartilage analysis) and no biomechanical testing. Both of which are the gold standard in the field. Additionally, the paper consistently refers to the rate of healing, but there is no time course to suggest that the rate is altered versus a simple change in the magnitude of healing. These problems along with the specific comments below should be addressed.

In describing Figure 1, the term "accelerated" is used to describe the effect of TNFa on fracture healing, but there is no time course showing that TNFa accelerates repair relative to control fractures. Can the authors comment on why the high dose of TNFa appears to have no effect? The time of analysis for 1C should be indicated in the text.

There is no data to support the statement: "Neutrophils were the predominant cell type present before day 3 while F4/80+ cells were the predominant cell type after day 3." Neutrophils were examined qualitatively at 24 hours after injury and macrophages were examined qualitatively at day 7. A quantitative analysis of both cell types at multiple time points is required to make the above statement. Fig. 1g does not show "from day 3 onwards," it shows F4/80+ cells at day 7 only.

The statement: "As neutrophils were the first cells to express TNF at the fracture site," is not supported by the analysis, because these were the only cell types examined (or at least the only cell type where data is presented). The data would need to show either the presence of other cell types (T, macrophages) that do not express TNFa, or the absence of these cells from the fracture site.

Supplemental Figure 1b is not convincing. The neutrophils should be stained with an antibody and quantified to show the treatment has depleted neutrophils.
The data clearly show that neutrophils can respond to TNFa and upregulate CCL2. Could there be other sources that are also affected by TNFa? Co-Immunostaining of the early fracture callus for CCL2 and Elastase (As in Supp Fig. 1F) would clearly show that neutrophils are the exclusive source of CCL2 at the fracture site.

The CT scan included in Fig 3 does not clearly show a non-union in the CCR2 antagonist-treated leg.

The statement: "This emphasizes some of the limitations of studying genetically altered animals, where compensatory pathways can be present." does not make sense to me as it is written, because presumably these compensatory pathways would be present in animals treated acutely as well. Can the authors please explain their point more clearly?

The data shown with the CCR2 antagonist suggest a large effect on fracture healing, but outcomes of fractures in CCR2-/- mice appears less dramatic. While this is mentioned, can the authors attempt to reconcile these differences? Does the antagonist have off-target effects? The authors refer to subtle phenotypes in GM mice that may influence interpretation of data, but they do not provide any substantial information upon which this statement is based.

In the paper by Xing et al, there appeared to be no difference in recruitment of neutrophils to the fracture site, yet here it is stated that the CCR2 axis also regulates neutrophil recruitment.

Bone has a unique set of macrophages called Ostealmacs, and work has described a role for these cells in fracture healing (Alexander et al, JBMR, 2011). A discussion of these cells should be present in this paper.

The Discussion stresses the importance of stromal stem cells in fracture healing. Can the authors be more specific? In normal fractures that vast majority of chondrocytes and osteoblasts are derived from the local periosteum and not the bone marrow or other sources (See Colnot, JBMR 2009). So, while TNFa may have osteogenic effects on stem cells derived from muscle of bone marrow, the relevance of these cells to fracture healing is unknown.

The Discussion needs significant editing. There are missing words (endothelial when it should state endothelial cells, the word message is used for mRNA, etc.).

Minor:
The quantitative data presented in the results should be put into tables rather than listed in the text. The differences will be more easily identifiable, but it does not follow from the earlier sentence which is about CCR2.

"However, they may also contribute to other aspects of fracture healing including the expression of osteogenic cytokines such as TNF and IL-6" does not make sense in the context of the preceding sentence. I think "they" is macrophages.

"have been shown to deleterious" should be "have been shown to be deleterious"

"While neutrophil have traditionally" should be "While neutrophils have traditionally"

Referee #2 (Comments on Novelty/Model System):

This study extend on prior findings that low local TNF alpha concentrations administered early in the first day after fracture improved the healing process.

In the current study these authors extend on this finding by further examining the mechanism by

which TNF alpha promotes fracture repair through the innate immune reaction.

In this study they show through both gain and loss studies that TNF alpha promotes early neutrophil infiltration into the fracture site.

They further show that depletion of neutrophils during the immediate period after injury decreased long term regain of fracture callus mineral content.

The role of CCL2 and CCR2 interaction via TNF alpha stimulation in the recruitment of monocytes was then assessed and shown to also be necessary for regain of fracture callus mineral content.

While the above studies all appeared to be very well carried out, the overall conclusion that either neutrophil depletion or loss of CCL2 CCR2 function effected fracture healing are inclusive.

1) Measurements by microCT analysis of simple mineral contents do not assess whether healing was delayed. It is also unclear what relative callus mineralization is measuring. Standardized microCT measurements inclusive of total callus volume TMD, TV/BV as well as BV should be presented.

2) Similarly only biomechanical assessments provide definitive measurements on fracture repair based on regain of normal mechanical function.

3) The actual effects on the mechanisms of endochondral bone formation that are being effected as it relates either to neutrophil deletion or due to blockade in CCL2/CCR2 activity needs to be presented.

Is there delay in bone and cartilage tissue formation or in progression of endochondral cartilage maturation? Are these tissues replaced with fibrous tissues?

Without these analysis, the underlying mechanism by which inference with the innate inflammatory activity effects fracture repair cannot be assessed. For example some measure of stem cell recruitment would be very informative to the current study.

Referee #2 (Remarks):

While this was a very interesting paper, and extended on these group's prior findings, their conclusions as to delay in fracture repair are only based on microCT analysis which is insufficient to prove impaired healing.

The studies do not provide mechanistic insight into how this particular perturbation of the early innate immune cells and functions altered endochondral bone formation. It has been speculated for a number a years that innate cell activities promote stem cell recruitment and/ or activation but the current studies do not provide much additional mechanistic insight.

Resubmission 18 August 2014

****** Reviewer's comments ******

Referee #1 (Comments on Novelty/Model System):

Technical quality: I rated this medium, because the majority outcome parameter to assess fracture healing is % callus mineralization. While this is a good measure, the analysis is not thorough. The gold standard includes bone and cartilage histomorphometry as well as biomechanical outcomes. I would not reject a paper without these additional data, but the authors are encouraged to be more thorough. They make comparisons to other research that are not in complete agreement with their data, and the authors attribute the differences to the fact that the previous research was performed in GM animals while there work was with small molecule inhibitors. Their explanation does not

make much sense to me as it is written.

Biomechanical testing is often considered as a gold standard in the assessment of bone quality in both intact and fractured bones. However, this is most reliable in larger bones, such as femora of rats. Recently, analysis of μCT parameters has been found to be a more sensitive method of evaluating murine fracture callus properties than biomechanical studies in part due to the greater amount of information that can be obtained to describe the structural and compositional properties of the callus with this non-destructive method (Nyman et al J Biomechanics 2009) (1). Furthermore, the sample size estimates for microCT parameters are much lower than those estimated using biomechanical measurements due to decreased variance and increased sensitivity.

We have now added in Supplemental Fig 2, which provides data on the total callus volume (TV) and mineralized callus volumes (BV). These have been shown reliable parameters of biomechanical strength and healing in fractured bone (O'Neill, Stutz et al Bone 2012). Of the methods investigated by our group, including 3- and 4-point bend testing, microCT analysis provided the most informative and sensitive method for assessing fracture healing in tibiae of mice, providing both qualitative and quantitative analyses of the fracture callus. We have included this in a limitations section at the end of Discussion section of the manuscript.

In accordance with the reviewer's comments, we have modified the discussion regarding GM animals.

Novelty: I rated this medium, because the main healing outcomes have been published previously (2011). The new data here is on the CCL2/CCR2 axis and macrophage involvement after TNFa treatment. I think this is more than an incremental advance, but this could have been included in the previous paper.

The data published in PNAS showed the importance of muscle as a reservoir of osteogenic precursors and that exogenous addition of TNF enhanced the recruitment and osteogenic recruitment of these cells. The body of work presented in our current manuscript was conducted *after* the PNAS publication and demonstrates the role and sequence of the innate immune response in fracture healing, which has not been shown previously. In particular we have demonstrated the importance of neutrophils and, as stated by the reviewer, the role of CCR2/CCL2 axis.

Medical Impact: I rated this high, because if the authors are correct a new treatment modality may be feasible.

We believe our data are important and clinically relevant as they demonstrate that manipulation of the early inflammatory response can lead to profound changes in the final healing outcome and hence represents a potential therapeutic target for the area of greatest clinical need, fractures in osteoporotic bone.

Referee #1 (Remarks):

This is a very interesting paper that describes the effect of TNFa on fracture healing, and indicates that the function of TNFa is mediated by influx of macrophages into the fracture site. Overall, this is an interesting story that could have important clinical impact. The data are restricted to analysis of % mineralization of the fracture callus, with no tissue level (bone vs. cartilage analysis) and no biomechanical testing. Both of which are the gold standard in the field.

Please see above.

Additionally, the paper consistently refers to the rate of healing, but there is no time course to suggest that the rate is altered versus a simple change in the magnitude of healing. These problems along with the specific comments below should be addressed.

In describing Figure 1, the term "accelerated" is used to describe the effect of TNFa on fracture healing, but there is no time course showing that TNFa accelerates repair relative to control fractures. Can the authors comment on why the high dose of TNFa appears to have no effect? The time of analysis for 1C should be indicated in the text.

We agree with the referee's comments and have changed the word 'accelerate' to 'augment'.

While our data show either augmentation or impairment of fracture healing at either day 14 or day 28 post-fracture, it is not possible to comment on whether the changes observed are due to a change in the rate or quality of fracture repair, or indeed, both. This is because the mice were sacrificed at each time point. For future investigations we will use vivo micro-CT imaging to follow the progression of fracture repair longitudinally over time, with the advantage that each mouse will serve as its own control to allow matched analyses. We have added this to the limitations section in the Discussion.

Re: high levels of TNF, we have added the following to the discussion:

Chronically high levels of TNF are known to impair fracture healing(2). It is interesting to note that addition of the highest dose of TNF in the early inflammatory window did not impair fracture healing at day 28. This may be due to the relatively short stimulation period of osteoclasts.

Time of analysis for the data shown in Fig 1c has now been indicated in the text as requested.

There is no data to support the statement: "Neutrophils were the predominant cell type present before day 3 while F4/80+ cells were the predominant cell type after day 3." Neutrophils were examined qualitatively at 24 hours after injury and macrophages were examined qualitatively at day 7. A quantitative analysis of both cell types at multiple time points is required to make the above statement. Fig. 1g does not show "from day 3 onwards," it shows F4/80+ cells at day 7 only.

We have added new Figures 2b and 2e, which quantify the neutrophils and monocytes at the fracture site at the different time points. Re: Fig 1g, we showed a representative image at day 7 and have reworded the appropriate section in the Results section.

The statement: "As neutrophils were the first cells to express TNF at the fracture site," is not supported by the analysis, because these were the only cell types examined (or at least the only cell type where data is presented). The data would need to show either the presence of other cell types (T, macrophages) that do not express TNFa, or the absence of these cells from the fracture site.

We used in situ hybridization to demonstrate the expression of TNF at the fracture site at the early time points. This then led us to examine the relevant regions in the adjacent histological slides and neutrophils and monocytes were the only inflammatory cells identified.

Supplemental Figure 1b is not convincing. The neutrophils should be stained with an antibody and quantified to show the treatment has depleted neutrophils.

We have added new Figures 2b and 2e which quantify the neutrophils and monocytes at the fracture site at the different time points.

The data clearly show that neutrophils can respond to TNFa and upregulate CCL2. Could there be other sources that are also affected by TNFa? Co-Immunostaining of the early fracture callus for CCL2 and Elastase (As in Supp Fig. 1F) would clearly show that neutrophils are the exclusive source of CCL2 at the fracture site.

CCL2 is known to be produced by a number of different cell types including endothelial cells, fibroblasts, vascular smooth muscle cells and bone marrow cells. Therefore, it is unlikely that neutrophils are the exclusive source of CCL2. However, as noted by the referee, we have shown that

neutrophils can respond to TNFa to upregulate CCL2 production, and we have also demonstrated the key roles of both neutrophils and CCL2 in fracture healing as depletion of each significantly impaired fracture healing (Figs 2c, d; 3a). We have reworded this section of the Discussion accordingly.

The CT scan included in Fig 3 does not clearly show a non-union in the CCR2 antagonist-treated leg.

Non-union is a failure of fracture repair and is characterized by aberrant fracture callus formation with no bridging across the fracture gap (3). We would respectfully submit that the microCT reconstructions show no bridging across the fracture gap.

The statement: "This emphasizes some of the limitations of studying genetically altered animals, where compensatory pathways can be present." does not make sense to me as it is written, because presumably these compensatory pathways would be present in animals treated acutely as well. Can the authors please explain their point more clearly?

We agree with the reviewer's comment and have modified the Discussion accordingly.

The data shown with the CCR2 antagonist suggest a large effect on fracture healing, but outcomes of fractures in CCR2-/- mice appears less dramatic. While this is mentioned, can the authors attempt to reconcile these differences? Does the antagonist have off-target effects? The authors refer to subtle phenotypes in GM mice that may influence interpretation of data, but they do not provide any substantial information upon which this statement is based.

We have added a section on the pharmacology of INCB3344 including a reference to support the limited off-target effects.

There are a number of genetically modified mice that have a disappointing lack of discernible phenotype due to functional redundancy in gene family members and we have reworded this section as well as provided an example. This is one possible explanation for the difference observed between CCR2 antagonism in wild type animals and CCR2-/- mice.

In the paper by Xing et al, there appeared to be no difference in recruitment of neutrophils to the fracture site, yet here it is stated that the CCR2 axis also regulates neutrophil recruitment.

Accurate quantification of cells in histological sections can be difficult. To overcome this limitation we used an air pouch model. We found that anti-CCL2 abrogated the recruitment of neutrophils and macrophages into the air pouch. Recent studies have also found that CCL2 is important for the recruitment of neutrophils during acute inflammation using a murine model of acute septic peritonitis(4, 5).

Bone has a unique set of macrophages called Ostealmacs, and work has described a role for these cells in fracture healing (Alexander et al, JBMR, 2011). A discussion of these cells should be present in this paper.

In accordance with the review's suggestion, we have added a discussion of osteomacs in the Discussion.

The Discussion needs significant editing. There are missing words (endothelial when it should state endothelial cells, the word message is used for mRNA, etc.).

These have now all been addressed.

Minor:

The quantitative data presented in the results should be put into tables rather than listed in the text. The differences will be more easily identifiable, but it does not follow from the earlier sentence which is about CCR2.

"However, they may also contribute to other aspects of fracture healing including the expression of osteogenic cytokines such as TNF and IL-6" does not make sense in the context of the preceding sentence. I think "they" is macrophages.

"have been shown to deleterious" should be "have been shown to be deleterious"

"While neutrophil have traditionally" should be "While neutrophils have traditionally"

These have all been addressed

Referee #2 (Comments on Novelty/Model System):

This study extend on prior findings that low local TNF alpha concentrations administered early in the first day after fracture improved the healing process.

In the current study these authors extend on this finding by further examining the mechanism by which TNF alpha promotes fracture repair through the innate immune reaction.

In this study they show through both gain and loss studies that TNF alpha promotes early neutrophil infiltration into the fracture site.

They further show that depletion of neutrophils during the immediate period after injury decreased long term regain of fracture callus mineral content.

The role of CCL2 and CCR2 interaction via TNF alpha stimulation in the recruitment of monocytes was then assessed and shown to also be necessary for regain of fracture callus mineral content.

While the above studies all appeared to be very well carried out, the overall conclusion that either neutrophil depletion or loss of CCL2 CCR2 function effected fracture healing are inclusive.

1) Measurements by microCT analysis of simple mineral contents do not assess whether healing was delayed. It is also unclear what relative callus mineralization is measuring. Standardized microCT measurements inclusive of total callus volume TMD, TV/BV as well as BV should be presented.

Supplemental Fig 2 has now been added. This shows the total callus volumes and mineralized callus volumes from which the normalized % callus mineralization values have been derived.

2) Similarly only biomechanical assessments provide definitive measurements on fracture repair based on regain of normal mechanical function.

Please see response above to a similar comment by reviewer 1.

3) The actual effects on the mechanisms of endochondral bone formation that are being effected as it relates either to neutrophil deletion or due to blockade in CCL2/CCR2 activity needs to be presented.

Is there delay in bone and cartilage tissue formation or in progression of endochondral cartilage maturation? Are these tissues replaced with fibrous tissues? Without these analysis, the underlying mechanism by which inference with the innate inflammatory activity effects fracture repair cannot be assessed. For example some measure of stem cell recruitment would be very informative to the current study.

Our study focuses on determining the key innate immune steps during early inflammation that contributes to the final healing outcome. Our histological images and microCT analyses support the conclusion that depletion of neutrophils lead to an immature callus formation whereas inhibition of CCR2 led to a non-union-like picture where there is an absence of cortical bridging. The reduced %

callus mineralization suggests a delay in endochondral cartilage maturation. We agree that it would be logical to further investigate this in greater detail and this will be the subject of a substantial follow-up study beyond the scope of the present manuscript.

Referee #2 (Remarks):

While this was a very interesting paper, and extended on these group's prior findings, their conclusions as to delay in fracture repair are only based on microCT analysis which is insufficient to prove impaired healing.

The studies do not provide mechanistic insight into how this particular perturbation of the early innate immune cells and functions altered endochondral bone formation. It has been speculated for a number a years that innate cell activities promote stem cell recruitment and/ or activation but the current studies do not provide much additional mechanistic insight.

The focus of this present study is to study the role of the innate immune response on fracture repair, which has not previously been described. In our view, the observation that manipulation of the early inflammatory events leads to dramatic effects on the downstream fracture healing process and final clinical outcome is novel and of huge clinical significance. Inflammation has often been associated with impaired repair processes, including bone healing as evidenced by the bone erosions seen in patients with rheumatoid arthritis and other inflammatory conditions(6). Hence much of osteoimmunology research has focused on the effect of acquired inflammatory pathways on bone destruction and osteoclastogenesis. However, at the same time, inflammation is also thought to be necessary in the initiation and orchestration of downstream processes that ultimately lead to tissue repair(7). There is little known about the role of the innate immune response in bone formation at present. The reviewer is correct to say that innate cell activities may promote stem cell recruitment and/or activation. We and others have recently shown that inflammatory cytokines including TNF can promote stem cell recruitment and osteogenic differentiation(8). In this follow-up study, we now show the role of the innate immune cells in promoting fracture repair, which has not previously been described, as well as how TNF accelerates fracture repair through upregulation of this pathway. The concept that early inflammation is an important event in fracture healing will represent a paradigm shift in the understanding of bone biology and how patients are managed in the clinical setting. We believe this paper will open up a large and fertile field for exploration of the downstream mechanisms by which innate immune cells lead to bone formation.

1. O'Neill KR, Stutz CM, Mignemi NA, Burns MC, Murry MR, Nyman JS, et al. Micro-computed tomography assessment of the progression of fracture healing in mice. Bone. 2012;50(6):1357-67. Epub 2012/03/29.

2. Alblowi J, Kayal RA, Siqueira M, McKenzie E, Krothapalli N, McLean J, et al. High levels of tumor necrosis factor-alpha contribute to accelerated loss of cartilage in diabetic fracture healing. The American journal of pathology. 2009;175(4):1574-85. Epub 2009/09/12.

3. Marsh D. Concepts of fracture union, delayed union, and nonunion. Clinical orthopaedics and related research. 1998(355 Suppl):S22-30. Epub 1999/01/26.

4. Matsukawa A, Hogaboam CM, Lukacs NW, Lincoln PM, Strieter RM, Kunkel SL. Endogenous monocyte chemoattractant protein-1 (MCP-1) protects mice in a model of acute septic peritonitis: cross-talk between MCP-1 and leukotriene B4. J Immunol. 1999;163(11):6148-54. Epub 1999/11/26.

5. Speyer CL, Gao H, Rancilio NJ, Neff TA, Huffnagle GB, Sarma JV, et al. Novel chemokine responsiveness and mobilization of neutrophils during sepsis. The American journal of pathology. 2004;165(6):2187-96. Epub 2004/12/08.

6. Hofbauer LC, Lacey DL, Dunstan CR, Spelsberg TC, Riggs BL, Khosla S. Interleukin-1beta and tumor necrosis factor-alpha, but not interleukin-6, stimulate osteoprotegerin ligand gene expression in human osteoblastic cells. Bone. 1999;25(3):255-9. Epub 1999/09/24.

7. Chan JK, Roth J, Oppenheim JJ, Tracey KJ, Vogl T, Feldmann M, et al. Alarmins: awaiting a clinical response. The Journal of clinical investigation. 2012;122(8):2711-9. Epub 2012/08/02.

8. Glass GE, Chan JK, Freidin A, Feldmann M, Horwood NJ, Nanchahal J. TNF-{alpha} promotes fracture repair by augmenting the recruitment and differentiation of muscle-derived stromal cells. Proceedings of the National Academy of Sciences of the United States of America. 2011. Epub 2011/01/07.

2nd Editorial Decision 19 September 2014

Thank you for the resubmission of your manuscript to EMBO Molecular Medicine. We have now heard back from the two referees whom we asked to evaluate your manuscript. I apologise for the wrong numbering, but referee 1 now was referee 2 in EMM-3511 review and referee 2 now was referee 1. I hope this will not be too confusing.

As you will see, they both still agree on the high interest of the topic and acknowledge that the revision does address some previously raised issues. However, the main problem found before remains and both referees clearly state that the biomechanical testing has to be done as this is the gold-standard method to show healing.

While the study cannot be considered for publication at this stage, we decided to give you a last chance to reply satisfactorily to the referees, with the understanding that the referees' concerns must be fully addressed and that acceptance of the manuscript would entail a second round of review.

Please note that it is EMBO Molecular Medicine policy to allow only a single round of revision and that, as acceptance or rejection of the manuscript will depend on another round of review, your responses should be as complete as possible.

I look forward to seeing a revised form of your manuscript within 3 months. As you go along, please let us know if you think you would need more time.

***** Reviewer's comments *****

Referee #1 (Comments on Novelty/Model System) [was referee #2 before]:

While the paper remains of great interest, the authors did not address the two major points of critique that had been raised in the first review.

Referee #1 (Remarks):

While the inclusion of additional micro CT data addresses to some degree that depletion of neutrophils or inhibition of the chemokine receptor CCR2 resulted in impaired fracture healing, these new data still do not provide much mechanistic insight into how neutrilphils or the actions of CCR2 mediate these negative effects.

Two specific requests as to the previous review were not addressed.

The first being if these neutrophils or inhibition of the chemokine receptor CCR2 effected actual regain of mechanical function. While micro CT data addresses mineral content and may qualitatively assess bridging, these data do not address the extent to which these changes affect function. This can only be addressed by mechanical testing.

The second was that the end points, which were used to assess healing by microCT, were carried out at relatively late times (14 and 28 days) post fracture. Since the observable formation of mineralized tissues comes about as the consequence of an endochondral process of bone formation, entailing both cartilage and bone cell differentiation, and mineral deposition that takes place both in cartilage and bone some assay of the content of these tissues needs to be carried out.

Since the treatments that block neutrophil CCR2 and TNF activity were carried out over the first eight days, this encompasses all of the early period in which initial osteogenic and chondrogenic differentiation is happening. It therefore is quite relevant to the mechanism as to how these treatments are effecting either the recruitment or early differentiation events of chondrocytes, osteoblasts, or both cell type' development.

It might also be informative to examine the numbers of progenitors of these cells using Sox9 or osterix immune or RNA expression assays at early time points.

Referee #2 (Comments on Novelty/Model System) [was referee #1 before]:

I chose medium for technical quality, becuase the author's still have not performed mechanical testing.

I chose medium for novelty, because there is a lot of work now on the influx of inflammatory cells into the fracture site. Further, these authros have published similar data in the past, and here they link CCL2/CCR2 to the outcomes.

This work has high medical impact!

Referee #2 (Remarks):

In the previous review the major concerns I raised were that biomechanical testing should be used to assess healing outcomes in fracture models in order to determine if an animal has healed. The authors have not adequately addressed this point. They have added more data on the mineralized callus, and they have not analyzed the cartilage component at all.

The statement that there are no F4/80 positive cells at the fracture site prior to day 3 does not agree with previously published data (See Alexander et al, JBMR 2010, and Wang et al, Bone 2013) that shows the timing of influx of neutrophils and macrophages to the fracture site. It is difficult to appreciate the co-localization of the TNF in the PMNs and the macrophages in the adjacent sections in Fig. 1f and 1g.

In Fig. 2c the top two panels (control animals) are in color, but the bottom two panels (treated animals) are in black and white. It appears that the top panels (color) is identical to the bottom two panels (black and white), and the labeling is wrong. Which is treated and control?

While I appreciate that the author's previous research suggests that muscle-derived mesenchymal cells may participate in bone healing, the healing shown here (Fig 2C) appears to be periosteal. Further, the role of the periosteum in healing of older animals is clearly established, and the statement of the muscle as the major contributor should be toned down.

1st Revision - authors' response 07 December 2014

We appreciate the constructive and encouraging comments by both reviewers. We will address each point individually below. However, we begin by addressing the issue of biomechanical testing, which is the major criticism of both reviewers.

We agree that biomechanical testing has traditionally been considered as the gold standard in the assessment of bone quality as these properties characterize the ability of repaired bone to endure physiological loads without re-fracturing. By measuring the callus strength relative to a control, it enables the effectiveness of intervention to be determined. However, it is also clear that biomechanical testing is severely limited by the large variation seen within experimental groups, particularly in small animals such as mice. A number of groups have published on the limitations of biomechanical testing in small animals. For example, Manigrosso et al found significant variability when bend testing fracture calluses in mice, with coefficient of variance calculations of over 30% (1). They calculated that to detect a 20% difference in peak torque at 3 weeks post fracture, 38 mice would be needed in each study group. This has also been confirmed in the latest and most comprehensive study of biomechanical testing in rodents by O'Neill et al (2).

The fracture callus is a complex irregular structure that consists of cartilaginous and mineralized matrix components. The relative proportions of these evolve throughout the process of callus maturation. The properties of each fracture callus are unique and determined by the precise configuration of the initial fracture (e.g. obliquity, degree of comminution), stability of fracture fixation and activity of the animal. The concept of bend testing relies on the premise that the object to be tested is a perfect cylinder and that the long bone is transversely isotropic i.e. the material has properties that are the same in two of the three axes(3). However, the fracture callus is never perfectly symmetrical and is orthotropic, i.e. possess properties that differ in each of three perpendicular axes. We found that three-point loading allowed consistent force-extension profiles to be generated when intact tibiae were bend-tested. However, due to the heterogeneous shape and configuration of the fracture callus, we found that three-point loading created high shear stresses at the site of the callus and pure bending could not be achieved. Whilst larger bones are subject to similar errors, the effects are greatly magnified in small bones such as the mouse tibia, which is less than 25mm in length. Under these conditions the morphology and composition of the callus at the precise point of placement of the upper loading point on the callus becomes the main factor that determines the force-extension profile obtained rather than the composition or size of the whole fracture callus. This accounted for the widely different force-extension profiles generated when we attempted to bend test our specimens. The only previous publication to report the results of mechanical testing of tibial fractures in mice was by our group in 2008(4). We were able to use this technique on groups of 10 mice or less as there was nearly a 3-fold difference in load to failure between groups. Our calculations show that in our current study over 100 samples would be required to detect a 10% difference where alpha is 0.05, power of test is 80%, SD 1.85 and 2-tailed test is used. The use of such large number of animals would be considered unethical and also would not have been approved by our local animal ethics committee, especially when microCT has been published to provide accurate and reliable surrogate measurements for fracture repair while requiring fewer animals(2).

MicroCT analysis has gained popularity over the past decade as it is a non-destructive and quantitative technique that enables a greater amount of information to be obtained which describes the structural and compositional properties of the callus(5). Consequently, it has also been found to be a more sensitive method of evaluating murine fracture callus properties than biomechanical studies. Furthermore, the sample size estimates for microCT parameters are much lower than those estimated using biomechanical measurements due to decreased variance and increased sensitivity. Another advantage of microCT is that it provides an insight into how an intervention affects fracture callus composition, particularly callus mineralization, which is not possible with biomechanical testing.

© EMBO

The use of microCT to assess fracture healing in pre-clinical studies is now well documented and has been validated by a number of groups(6-15). These studies have demonstrated good agreement among CT, radiology, histology-derived measures of healing and biomechanical testing. Direct comparisons of CT and standard radiographic analyses have found that CT yields comparable or better predictions of callus compressive strength(10) and torsional strength and stiffness(8, 11), more definitive indicators of healing progression(6) and of non-union(16). Morgan et al studied microCT parameters and callus mechanical properties of murine femoral fracture calluses using torsional strength as the main study outcome and found that the best predictors of fracture callus mechanical properties were microCT-derived measures of callus structure and composition(17). Markel et al 1990 used non-invasive techniques to assess calluses in canine tibiae and found quantification of BMD by micro CT and single photon absorptiometry correlated with torsional strength and stiffness(18). Furthermore, Tiedeman et al 1990 found the density of new bone formation within the fracture gap correlated strongly with bending rigidity(19). Nyman et al. has published the most comprehensive analysis of biomechanical testing versus CT in the literature(5). They studied healing of femoral fractures in rats and looked for correlation between and callus strength on biomechanical testing and surrogate measurements on CT. Femoral fractures were treated locally with different doses of lovastatin in order to generate a range of healing rates, (n=8-12 per group). Similar to our study, the animals were sacrificed at one time point, 4 weeks post-op. They found that larger calluses were weaker than smaller calluses. Furthermore, the density of the mineralized tissue within the callus strongly correlated with callus strength(5). Other authors have also found that the mineralization of the fracture callus is a good predictor of strength, for example Shelfelbine et al 2005 observed a significant positive correlation between minimum BMD and torsional rigidity(20).

In conclusion, microCT provides a useful, reliable, appropriate and ethical way to assess fracture repair in preclinical studies.

***** Reviewer's comments *****

Referee #1 (Comments on Novelty/Model System) [was referee #2 before]:

While the paper remains of great interest, the authors did not address the two major points of critique that had been raised in the first review.

Referee #1 (Remarks):

While the inclusion of additional micro CT data addresses to some degree that depletion of neutrophils or inhibition of the chemokine receptor CCR2 resulted in impaired fracture healing, these new data still do not provide much mechanistic insight into how neutrophils or the actions of CCR2 mediate these negative effects.

Two specific requests as to the previous review were not addressed.

The first being if these neutrophils or inhibition of the chemokine receptor CCR2 effected actual regain of mechanical function. While micro CT data addresses mineral content and may qualitatively assess bridging, these data do not address the extent to which these changes affect function. This can only be addressed by mechanical testing.

Please see above regarding the use of microCT and not mechanical testing

The second was that the end points, which were used to assess healing by microCT, were carried out at relatively late times (14 and 28 days) post fracture. Since the observable formation of mineralized tissues comes about as the consequence of an endochondral process of bone formation, entailing both cartilage and bone cell differentiation, and mineral deposition that takes place both in cartilage and bone some assay of the content of these tissues needs to be carried out.

Since the treatments that block neutrophil CCR2 and TNF activity were carried out over the first eight days, this encompasses all of the early period in which initial osteogenic and chondrogenic differentiation is happening. It therefore is quite relevant to the mechanism as to how these treatments are effecting either the recruitment or early differentiation events of chondrocytes, osteoblasts, or both cell type' development.

It might also be informative to examine the numbers of progenitors of these cells using Sox9 or osterix immune or RNA expression assays at early time points.

We agree that elucidating the relative contribution of the different cell types to the fracture callus would be an interesting study. However, the focus of our article is rather different: to investigate whether the early inflammatory response contributes to the final fracture healing outcome in vivo. Our data show that manipulation of the early inflammatory environment does indeed affect fracture healing. We have provided data on the mineralization of fracture callus as our main outcome as well as added total callus volume as well as bone volume within callus in Supplemental Fig 2.

Referee #2 (Comments on Novelty/Model System) [was referee #1 before]:

I chose medium for technical quality, because the author's still have not performed mechanical testing.

Please see above regarding mechanical testing

I chose medium for novelty, because there is a lot of work now on the influx of inflammatory cells into the fracture site. Further, these authors have published similar data in the past, and here they link CCL2/CCR2 to the outcomes.

We are puzzled by the reviewers suggesting that we have published similar data in the past. Our previous publication was on the role of inflammatory cytokines, especially TNF, on promoting migration and osteogenic differentiation of muscle-derived mesenchymal stromal cells in vitro. There were limited data on the in vivo effects of TNF on fracture healing. Here we:

1) characterize the in vivo effects of local TNF on fracture repair, including timing and dose response
2) demonstrate that anti-TNF and rm-IL10 inhibit fracture repair in vivo
3) show the expression of TNF by neutrophils and monocytes during the early innate inflammatory window following fracture injury
4) show that TNF leads to enhanced recruitment of neutrophils and monocytes/macrophages in the fracture environment
5) show inhibition of fracture repair in vivo by neutrophil depletion
6) demonstrate that role of the TNF/CCL2/CCR2 axis in fracture repair, and
7) show that additional TNF accelerates fracture repair in osteoporotic mice.

All these findings are new. To our knowledge, this will be the first published report to show that manipulation of the early inflammatory response affects the final fracture outcome. Most of the work in the field focuses on the role of the acquired immune response on bone. There is a paucity of research that focuses on the role of the innate immune response on fracture healing, While we have previously published the potential contribution of TNF in fracture healing, it is a substantial novel step to show that it is the innate immune response that is key to enable the fracture healing pathway and that TNF acts via the CCL2/CCR2 pathway.

This work has high medical impact! -

Thank you, we agree!!

Referee #2 (Remarks):

In the previous review the major concerns I raised were that biomechanical testing should be used to assess healing outcomes in fracture models in order to determine if an animal has healed. The authors have not adequately addressed this point. They have added more data on the mineralized callus, and they have not analyzed the cartilage component at all.

Our study focuses on determining the key innate immune steps during early inflammation that contributes to the final healing outcome. Our histological images and microCT analyses support the conclusion that depletion of neutrophils lead to an immature callus formation whereas inhibition of CCR2 led to a non-union-like picture with an absence of cortical bridging. The reduced % callus mineralization suggests a delay in endochondral cartilage maturation. We agree that it would be logical to further investigate this in greater detail and this will be the subject of a substantial follow-up study beyond the scope of the present manuscript.

The statement that there are no F4/80 positive cells at the fracture site prior to day 3 does not agree with previously published data (See Alexander et al, JBMR 2010, and Wang et al, Bone 2013) that shows the timing of influx of neutrophils and macrophages to the fracture site. It is difficult to appreciate the co-localization of the TNF in the PMNs and the macrophages in the adjacent sections in Fig. 1f and 1g.

We would respectfully submit that our observation that no F4/80 positive cells were present at the fracture site in our fracture model does not conflict with previously published data. Alexander et al identified F4/80+ cells in bone-lining tissues (periosteum) which were intimately associated with osteoblasts on new woven bone surfaces (Alexander et al JBMR 2011). In contrast to Alexander et al, our murine fracture model involves stripping of the periosteum over a distance of 8 mm around the fracture site and is a model of endochondral, not intramembranous, healing. This is very important as the predominant form of healing seen in adult humans is endochondral. The earliest time point at which Alexander et al looked for F4/80+ cells was day 4 whereas we studied multiple time points over the period 3 hours to 7 days post-fracture. Using our model, we found that F4/80 positive cells appeared at the fracture site from day 3 onwards. Thus our observations do not conflict with those of Alexander et al.

Re: Figs 1f and 1g, the sections are 3 μm thick and adjacent sections were used. The white signal on the ISH images represent message for TNF and the dark brown stain on the H&E images stains for neutrophils in 1f and F4/80+ cells in 1g. The legend has been amended to clarify this.

In Fig. 2c the top two panels (control animals) are in color, but the bottom two panels (treated animals) are in black and white. It appears that the top panels (color) is identical to the bottom two panels (black and white), and the labeling is wrong. Which is treated and control?

Apologies and thank you for spotting this. The left column showed representative images of a control and the right column, the treatment (neutrophil depleted) group. We have now rearranged these images so that the top row represents the controls and bottom row the treatment group, consistent with Fig 2b for clarity. The B&W images were added to provide clear labeling of the anatomical structures to help readers. We have clarified this in the text.

While I appreciate that the author's previous research suggests that muscle-derived mesenchymal cells may participate in bone healing, the healing shown here (Fig 2C) appears to be periosteal. Further, the role of the periosteum in healing of older animals is clearly established, and the statement of the muscle as the major contributor should be toned down.

Regarding Fig 2C, we respectfully submit that the healing shown here is not periosteal. Our murine tibial fracture model, as mentioned above and detailed in the Methods section, involves stripping of the periosteum over a distance of 8 mm around the fracture site. This ensures that fracture repair occurs by endochondral rather than intramembranous healing as the murine periosteum is high active. This model was specifically designed to mimic the endochondral fracture healing seen in adult human long bones particularly following high energy fractures, where the periosteum is often stripped and muscle is likely a major contributor of osteoprogenitors(21, 22). We agree that the

periosteum contributes substantially to fracture healing in the skeletally immature human and adult mice, but this is not the case in adult humans (23).

1. Manigrasso MB, O'Connor JP. Characterization of a closed femur fracture model in mice. Journal of orthopaedic trauma. 2004;18(10):687-95. Epub 2004/10/28.

2. O'Neill KR, Stutz CM, Mignemi NA, Burns MC, Murry MR, Nyman JS, et al. Micro-computed tomography assessment of the progression of fracture healing in mice. Bone. 2012;50(6):1357-67. Epub 2012/03/29.

3. Turner CH, Burr DB. Basic biomechanical measurements of bone: a tutorial. Bone. 1993;14(4):595-608. Epub 1993/07/01.

4. Harry LE, Sandison A, Paleolog EM, Hansen U, Pearse MF, Nanchahal J. Comparison of the healing of open tibial fractures covered with either muscle or fasciocutaneous tissue in a murine model. Journal of orthopaedic research : official publication of the Orthopaedic Research Society. 2008;26(9):1238-44. Epub 2008/04/12.

5. Nyman JS, Munoz S, Jadhav S, Mansour A, Yoshii T, Mundy GR, et al. Quantitative measures of femoral fracture repair in rats derived by micro-computed tomography. Journal of biomechanics. 2009;42(7):891-7. Epub 2009/03/14.

6. Grigoryan M, Lynch JA, Fierlinger AL, Guermazi A, Fan B, MacLean DB, et al. Quantitative and qualitative assessment of closed fracture healing using computed tomography and conventional radiography. Academic radiology. 2003;10(11):1267-73. Epub 2003/11/25.

7. Schnarkowski P, Redei J, Peterfy CG, Weidenmaier W, Mutschler W, Arand M, et al. Tibial shaft fractures: assessment of fracture healing with computed tomography. Journal of computer assisted tomography. 1995;19(5):777-81. Epub 1995/09/01.

8. den Boer FC, Bramer JA, Patka P, Bakker FC, Barentsen RH, Feilzer AJ, et al. Quantification of fracture healing with three-dimensional computed tomography. Archives of orthopaedic and trauma surgery. 1998;117(6-7):345-50. Epub 1998/08/26.

9. den Boer FC, Bramer JA, Blokhuis TJ, Van Soest EJ, Jenner JM, Patka P, et al. Effect of recombinant human osteogenic protein-1 on the healing of a freshly closed diaphyseal fracture. Bone. 2002;31(1):158-64. Epub 2002/07/12.

10. Jamsa T, Koivukangas A, Kippo K, Hannuniemi R, Jalovaara P, Tuukkanen J. Comparison of radiographic and pQCT analyses of healing rat tibial fractures. Calcified tissue international. 2000;66(4):288-91. Epub 2000/04/01.

11. Augat P, Merk J, Genant HK, Claes L. Quantitative assessment of experimental fracture repair by peripheral computed tomography. Calcified tissue international. 1997;60(2):194-9. Epub 1997/02/01.

12. Luppen CA, Blake CA, Ammirati KM, Stevens ML, Seeherman HJ, Wozney JM, et al. Recombinant human bone morphogenetic protein-2 enhances osteotomy healing in glucocorticoid-treated rabbits. Journal of bone and mineral research : the official journal of the American Society for Bone and Mineral Research. 2002;17(2):301-10. Epub 2002/01/29.

13. Malizos KN, Papachristos AA, Protopappas VC, Fotiadis DI. Transosseous application of low-intensity ultrasound for the enhancement and monitoring of fracture healing process in a sheep osteotomy model. Bone. 2006;38(4):530-9. Epub 2005/12/20.

14. Li G, Bunn JR, Mushipe MT, He Q, Chen X. Effects of pleiotrophin (PTN) over-expression on mouse long bone development, fracture healing and bone repair. Calcified tissue international. 2005;76(4):299-306. Epub 2005/04/07.

15. Komatsubara S, Mori S, Mashiba T, Nonaka K, Seki A, Akiyama T, et al. Human parathyroid hormone (1-34) accelerates the fracture healing process of woven to lamellar bone replacement and new cortical shell formation in rat femora. Bone. 2005;36(4):678-87. Epub 2005/03/23.

16. Kuhlman JE, Fishman EK, Magid D, Scott WW, Jr., Brooker AF, Siegelman SS. Fracture nonunion: CT assessment with multiplanar reconstruction. Radiology. 1988;167(2):483-8. Epub 1988/05/01.

17. Morgan EF, Mason ZD, Chien KB, Pfeiffer AJ, Barnes GL, Einhorn TA, et al. Micro-computed tomography assessment of fracture healing: relationships among callus structure, composition, and mechanical function. Bone. 2009;44(2):335-44. Epub 2008/11/18.

18. Markel MD, Wikenheiser MA, Morin RL, Lewallen DG, Chao EY. Quantification of bone healing. Comparison of QCT, SPA, MRI, and DEXA in dog osteotomies. Acta orthopaedica Scandinavica. 1990;61(6):487-98. Epub 1990/12/01.

19. Tiedeman JJ, Lippiello L, Connolly JF, Strates BS. Quantitative roentgenographic densitometry for assessing fracture healing. Clinical orthopaedics and related research. 1990(253):279-86. Epub 1990/04/01.

20. Shefelbine SJ, Simon U, Claes L, Gold A, Gabet Y, Bab I, et al. Prediction of fracture callus mechanical properties using micro-CT images and voxel-based finite element analysis. Bone. 2005;36(3):480-8. Epub 2005/03/22.

21. Liu R, Schindeler A, Little DG. The potential role of muscle in bone repair. Journal of musculoskeletal & neuronal interactions. 2010;10(1):71-6. Epub 2010/03/02.

22. Chan JK, Harry L, Williams G, Nanchahal J. Soft-tissue reconstruction of open fractures of the lower limb: muscle versus fasciocutaneous flaps. Plastic and reconstructive surgery. 2012;130(2):284e-95e. Epub 2012/07/31.

23. O'Driscoll SW, Saris DB, Ito Y, Fitzimmons JS. The chondrogenic potential of periosteum decreases with age. Journal of orthopaedic research : official publication of the Orthopaedic Research Society. 2001;19(1):95-103. Epub 2001/05/03.

3rd Editorial Decision 12 January 2015

Thank you for the submission of your revised manuscript to EMBO Molecular Medicine. We have now received the enclosed report from the referee who was asked to re-assess it. As you will see the reviewer is now globally supportive and I am pleased to inform you that we will be able to accept your manuscript pending some editorial amendments.

Please submit your revised manuscript within two weeks. I look forward to seeing a revised form of your manuscript as soon as possible.

***** Reviewer's comments *****

Referee #2 (Remarks):

In my previous review, I indicated three minor issues: 1) to address the issue of the lack of mechanical testing, 2) to resolve the discrepancy between published data on the influx of macrophages to the fracture site beginning prior to day 3 and the failure to observe macrophages prior to day 3 here, and 3) to tone down the language regarding the role of the muscle in bone fracture healing in this model. None of these have been addressed in the manuscript.

1. The authors have provided an informative response to the issue of biomechanical testing. It would benefit the field to place some of this information into the Discussion where this is addressed, particularly since biomechanical testing is thought to be the gold-standard.

2. The failure to detect F4/80+ cells prior to day 3 remains a problem, because it does not agree with

previous studies. While the data shown in Alexander et al, does not illustrate macrophages prior to day 4, the authors indicate that there is an inflammatory reaction during days 1-3 (data not shown) and they state: "inflammatory macrophages, were present within the bone injury site and persisted throughout the healing time course." Work by Wang et al (Bone, 2013), which used models of intramembranous and endochondral ossification, shows F4/80+ cells at the fracture site by day 2 after injury using both FACS and immunohistochemistry. Previous work by Hankemeier et al, (JOR 2001) describes macrophages at the fracture site as early as day 1 after injury.

3. The role of the periosteum during fracture healing in aged animals is well documented (Lu et al, 2005 for example). The discussion of the contribution of muscle to bone healing should be toned down. The work by Liu et al, shows a modest contribution of MyoD positive cells to the cartilage callus after periosteal stripping, but there are a large number of cells that are not derived from MyoD-positive cells. This is the only experimental work that I am aware of showing a direct contribution of muscle-derived cells to the callus. The work in Glass et al, and the current work, do not contain a lineage analysis to show the origin of the cells, and the histology images appear to show a callus derived from the periosteum, particularly the anterior bone that is not adjacent to the muscle (Fig. 2 C).

© EMBO
